# Improving outcomes for care partners of persons with traumatic brain injury: Protocol for a randomized control trial of a just-in-time-adaptive self-management intervention

**DOI:** 10.1371/journal.pone.0268726

**Published:** 2022-06-09

**Authors:** Noelle E. Carlozzi, Angelle M. Sander, Sung Won Choi, Zhenke Wu, Jennifer A. Miner, Angela K. Lyden, Christopher Graves, Srijan Sen

**Affiliations:** 1 Department of Physical Medicine and Rehabilitation, University of Michigan, Ann Arbor, Michigan, United States of America; 2 H. Ben Taub Department of Physical Medicine and Rehabilitation, Baylor College of Medicine/Harris Health System, Houston, Texas, United States of America; 3 Brain Injury Research Center, TIRR Memorial Hermann, Houston, Texas, United States of America; 4 Department of Pediatrics, University of Michigan, Ann Arbor, Michigan, United States of America; 5 Department of Biostatistics, School of Public Health, University of Michigan, Ann Arbor, Michigan, United States of America; 6 Michigan Institute for Data Science, University of Michigan, Ann Arbor, Michigan, United States of America; 7 Clinical Trials Support Office, University of Michigan, Ann Arbor, Michigan, United States of America; 8 Department of Psychiatry, University of Michigan, Ann Arbor, Michigan, United States of America; PLOS: Public Library of Science, UNITED KINGDOM

## Abstract

Informal family care partners of persons with traumatic brain injury (TBI) often experience intense stress resulting from their caregiver role. As such, there is a need for low burden, and easy to engage in interventions to improve health-related quality of life (HRQOL) for these care partners. This study is designed to evaluate the effectiveness of a personalized just-in-time adaptive intervention (JITAI) aimed at improving the HRQOL of care partners. Participants are randomized either to a control group, where they wear the Fitbit^®^ and provide daily reports of HRQOL over a six-month (180 day) period (without the personalized feedback), or the JITAI group, where they wear the Fitbit^®^, provide daily reports of HRQOL and receive personalized self-management pushes for 6 months. 240 participants will be enrolled (n = 120 control group; n = 120 JITAI group). Outcomes are collected at baseline, 1-, 2-, 3-, 4-, 5- & 6-months, as well as 3- and 6-months post intervention. We hypothesize that the care partners who receive the intervention (JITAI group) will show improvements in caregiver strain (primary outcome) and mental health (depression and anxiety) after the 6-month (180 day) home monitoring period. Participant recruitment for this study started in November 2020. Data collection efforts should be completed by spring 2025; results are expected by winter 2025. At the conclusion of this randomized control trial, we will be able to identify care partners at greatest risk for negative physical and mental health outcomes, and will have demonstrated the efficacy of this JITAI intervention to improve HRQOL for these care partners.

**Trial registration**: ClinicalTrial.gov NCT04570930; https://clinicaltrials.gov/ct2/show/NCT04570930.

## Introduction

Illness impacts the entire family, and the complete picture of human disease is a collage of the experiences of both the affected patient and family care partner(s) [1]. As a society, responsibility for addressing these needs has always been placed on family care partners, who face an enormous and growing burden providing care to a loved one while trying to maintain their own health and well-being [2].

Traumatic brain injury (TBI) is a particularly challenging condition for family care partners due to the unexpected burden of providing prolonged supportive care at home once the survivor has been discharged from the hospital or rehabilitation facility. Care partners of persons with TBI must not only learn to cope with dramatic changes in their loved one’s health and functional abilities, but they also may feel ill-equipped to provide the requisite level of care.

Care partners of persons with TBI commonly experience problems with physical and mental HRQOL as a result of the caregiver role. Clinical interventions that focus on the self-care of care partners are critically important to improving health outcomes for both care partners and persons with TBI. Care partners may recognize the importance of self-care; however, the time demands of caring for an individual with TBI, as well as the associated physical and emotional toll, make it difficult for these individuals to prioritize their own self-care. Our preliminary work has highlighted the difficulty that care partners have engaging in positive health behaviors (e.g., physical activity or exercise) [3, 4]. This is especially unfortunate given recently published data in TBI that shows increases in physical activity are associated with improvements in anxiety and depression [5]. In fact, care partners of persons with TBI often report forgetting to engage in health management strategies focused on their own well-being [6]. Self-management programs (i.e., programs focused on medical/behavioral management, role management, and emotional management [7]) provide a potential avenue for fostering care partner health, yet those that have been examined in care partners of persons with TBI are burdensome and costly, and have limited impact on HRQOL in these individuals [7–11].

Although mobile health interventions offer the advantages of convenience, reach and scalability to promote health and well-being (including just-in-time adaptive interventions [JITAIs]), the clinical utility of this type of intervention in care partners of persons with TBI remains untested. We will address this critical gap in the literature by using passive data (i.e., physical activity and sleep data derived from a wrist-worn Fitbit^®^) and real-time assessments of HRQOL to identify and provide support to care partners of persons with TBI at the greatest risk for negative outcomes. We will also assess the effectiveness of a low-cost, low-burden intervention (the JITAI) designed to improve HRQOL outcomes in these care partners. We will examine the efficacy of the JITAI to improve self-reported mental HRQOL (including caregiver strain, depression and anxiety) in care partners of persons with TBI.

## Methods

### Participants and setting

240 care partners of persons with TBI will participate in this study. Participant recruitment and enrollment will take place at two data collection sites–(1) the University of Michigan and (2) Baylor College of Medicine/TIRR Memorial Hermann. Given that the COVID-19 pandemic occurred immediately prior to the start of this trial, the study was designed to allow participation to be completely virtual.

#### Inclusion criteria

Care partners must be at least 18 years old, be able to read and understand English and be caring for an adult (age 18 or above) with a medically documented TBI who sustained their TBI at age 16 or older. Care partners must be providing some form of care to the person with TBI. Specifically, care partners must indicate a response ≥1 to the following question: “On a scale of 0–10, where 0 is ‘no assistance’ and 10 is ‘assistance with all activities,’ how much assistance does the person you care for require from you to complete activities of daily living due to problems resulting from his/her TBI? Activities could consist of personal hygiene, dressing and undressing, housework, taking medications, managing money, running errands, shopping for groceries or clothing, transportation, meal preparation and cleanup, remembering things, etc.” Care partners of persons with TBI must be caring for an individual who is ≥1 year post-injury (to allow for stabilization of functioning in the person with TBI [12–23]) and meet TBI Model Systems inclusion criteria [24] for complicated mild, moderate or severe TBI. Specifically, complicated mild TBI is defined as an emergency room GCS score of 13–15 with positive findings on neuroimaging. Moderate to severe TBI is defined by at least one of the following: 1) post traumatic amnesia greater than 24 hours, 2) trauma related intracranial neuroimaging abnormalities, 3) loss of consciousness greater than 30 minutes not due to sedation or intoxication, 4) Glasgow Coma Scale in the emergency room of less than 13, not due to intubation, sedation or intoxication. Caregivers must also have access to the necessary resources for participating in a technology-based intervention and be willing to use their personal mobile device (e.g., smartphone, tablet) for this study, be willing to download the study app (CareQOL) and Fitbit^®^ app and be willing to complete all study assessments.

#### Exclusion criteria

Professional paid caregivers (e.g., home health aide) are excluded from this study.

### Recruitment and screening

Sites will recruit participants primarily through their established medical and TBI clinics, participant registries (both TBI- and care partner-specific registries), and clinical databases. Study participants will also be recruited through local TBI and care partner support groups, from organizations like the Brain Injury Association, and through targeted Facebook ads. Potential participants will be recruited directly or through the person they are caring for. Individuals who are interested in participating will be encouraged to ask questions about the study and their participation, and if they opt to enroll, they will provide informed consent prior to completing any study assessments.

### Study design

This behavioral trial will use a two-arm randomized controlled design. Study participation will involve a baseline assessment followed by a 10-day run-in period then a six-month home-monitoring period (in which the intervention will be administered to the JITAI group). After the completion of the 6-month home monitoring period, two post-intervention assessments will be administered at 9- and 12-months. Care partners will be block randomized at a 1:1 rate to the *control* or the *JITAI* group (e.g., n = 120 control; n = 120 JITAI). For both groups, the baseline assessment will include the completion of several self-report measures (including the TBI-CareQOL HRQOL measures) and instructions for the home monitoring period. During the home monitoring period, both groups will wear the Fitbit^®^ (e.g., Inspire) to continuously monitor physical activity and sleep, and both groups will complete real-time ratings of HRQOL (i.e., stress, worry and sadness) using the *CareQOL app*. Each participant will be prompted by a push notification in a five-hour window (based on participant preference) from the app to answer the questions. Care partners randomized to the JITAI group will have a 50/50 chance of receiving personalized “pushes” each day (through the *CareQOL app*). If randomized to receive the intervention message that day, the push notification will be delivered five hours after the time selected to receive the daily HRQOL questions. This notification can be viewed quickly on the phone’s lock screen, or participants can open the app to view the notification on the app’s home screen, where it stays until the next intervention message is sent. This allows the participant to choose not to engage with the notification (e.g., ignore it) at the time it is sent if it is inconvenient since they can return to it later.

**Fig 1:**
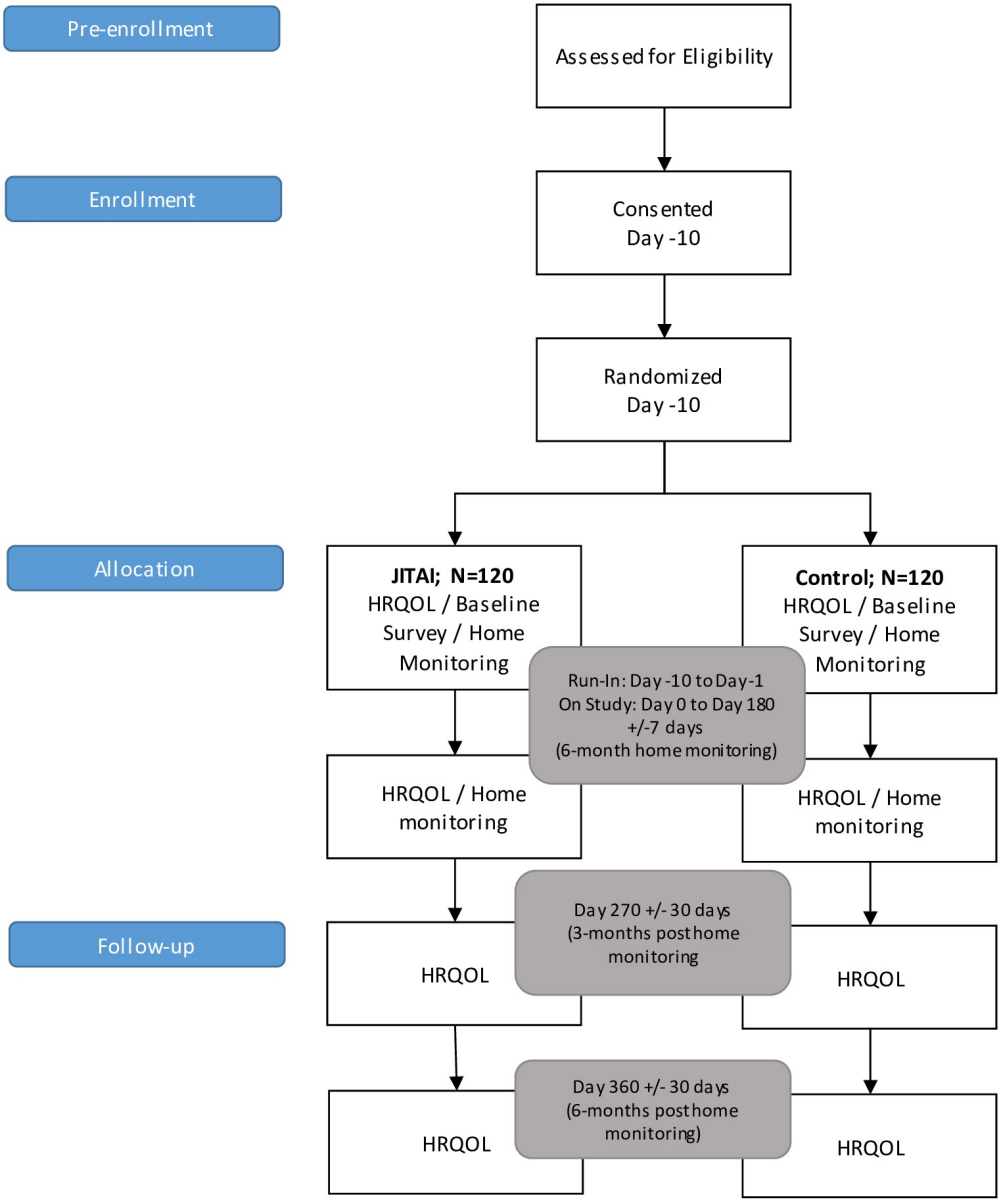
Study schema. Consort flow diagram.

Care partners in both groups will complete a portion of the TBI-CareQOL baseline measures at the end of each month during the home monitoring period, as well as at 9- and 12-months (i.e., 3- and 6-months post intervention). The study design is detailed in Fig 1.

### Randomization

Blocked randomization will be used to limit bias and achieve an equal distribution of participants to the control and treatment arms. A randomization list will be generated for each site and the study statistician will oversee randomization. The participant will be randomized once they are deemed eligible and have provided informed consent (i.e., at enrollment prior to baseline data collection). The study coordinator/research assistant who consented the participant will use their site’s randomization list to assign the participant to the correct study arm.

### Intervention

**Fig 2:**
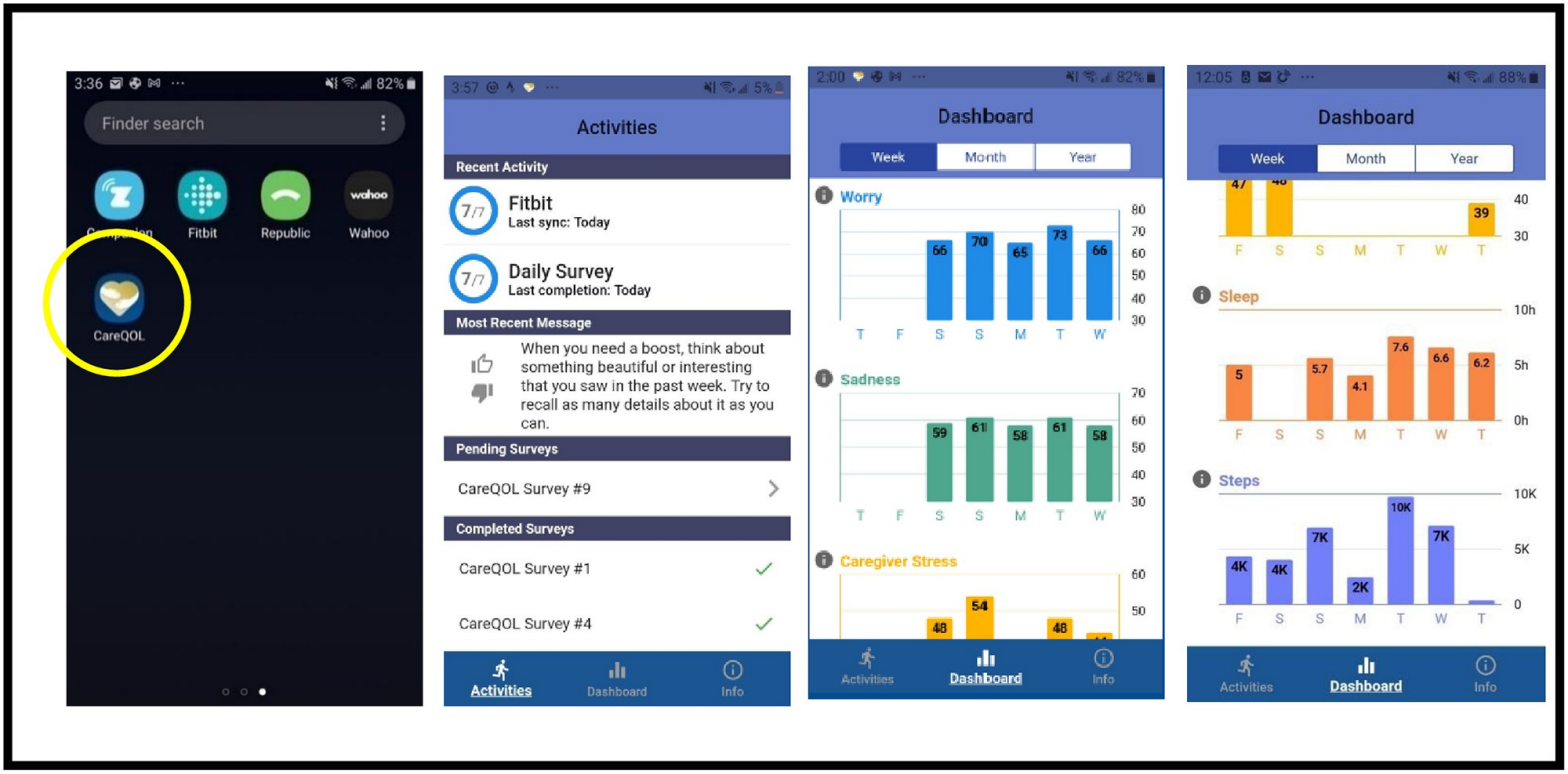
CareQOL app. Screen shots of the different elements of the CareQOL App.

Just-in-time adaptive intervention (JITAI) is a mobile health behavior-change approach that operationalizes the selection and delivery of personalized mobile device intervention strategies based on real-time data collection. In this study, a study-specific app (*CareQOL app*) will integrate sensor data from a Fitbit^®^ (on physical activity and sleep) with real-time self-report ratings of HRQOL (caregiver strain, depression, anxiety) to inform the JITAI. The *CareQOL app* will provide standard feedback to all study participants consisting of graphical displays of individual behaviors over time that users will be able to “pull” from the app at any time (i.e., stress, worry, sadness, sleep, and activity level graphs can be viewed at the users’ discretion 24/7; Fig 2). Among caregivers that are randomized to the intervention group, the JITAI will deliver personalized messages via the app ~50% the days during the 6-month intervention period; each day, the user has a 50/50 chance of receiving a feedback message.

The JITAI push notifications are aimed at promoting healthy behaviors (physical activity and good sleep hygiene) and improving mood (anxiety, depression, caregiver strain). Push notifications were broadly based on Behavioral Activation (BA) theory, which posits that negative life events (e.g., difficult interactions between the care partner and care-recipient, increased care partner stress due to caregiver role overload, etc.) trigger negative emotional responses (e.g., depression, anxiety, etc.) that lead to unhealthy behavioral patterns (e.g., poor sleep, decreased exercise, social withdrawal), which starts the cycle all over again [25]. BA (including BA delivered via text messaging) has been shown to be effective for treating both anxiety and depression, as “pure” constructs as well as for persons who are experiencing a mixture of the two [5, 26–30].

Specific “pushes” for this study were adapted from those that have been tested in our previous work [31, 32] using an iterative process that involved expert review and stakeholder input. Existing “pushes” (from our previous work) were reviewed for appropriateness for a care partner population and to identify gaps in content coverage. Prompts were added, deleted, or modified to ensure relevance to care partners. The adapted text was then reviewed by key stakeholders (care partners of persons with TBI, advocates for care partners of persons with TBI, and healthcare providers) and additional modifications were made resulting in a final pool of 411 prompts. Some messages use participants’ data directly in the messages (e.g., “You walked an average of 8,120 steps this week”), and most messages are designed to be personalized based on data (e.g., someone with low steps will get a different message than someone with medium steps than someone with high steps). Messages are comprised of one or more of the following different types: 1) Data feedback; 2) Facts; 3) Tips; and 4) Support. If receiving a notification, the message is randomly drawn from this pool of messages. Randomization of the days the participants receive messages and the message the participant receives from the pool will be done through the *CareQOL app*.

### Outcomes

The primary objective of this study is to examine the efficacy of the JITAI to improve self-reported caregiver strain in care partners of persons with traumatic brain injury (TBI). Table 1 provides a detailed summary of the study assessments and Fig 1 details the schedule of events. Briefly, participants will complete a baseline survey, a 10-day run-in period that will include the completion of 3 HRQOL items each day, a 6-month (~180 day) home monitoring period where participants wear the Fitbit^®^ and complete daily and monthly HRQOL assessments, and two follow-up assessments. The baseline assessment includes the completion of a series of surveys designed to characterize the sample with respect to demographics, caregiving experiences, and usual coping or management strategies, as well as a large battery of HRQOL measures that have been previously developed and validated for use in this population (i.e., the TBI-CareQOL measurement system [33–36]). The 10-day run-in period provides time to allow for shipping the Fitbit^®^ to participant’s homes, and gives the participant the opportunity to familiarize themselves with the study technology (Fitbit^®^, *CareQOL app*), and includes three real-time daily questions about HRQOL (that are administered as a computer adaptive test over the course of the week). During the 6-month home-monitoring period, all participants will continue to answer three daily HRQOL questions, as well as slightly longer HRQOL surveys that are administered at the end of each month. HRQOL surveys are also administered at 3- and 6-months following the home monitoring period.

**Table 1:** Outcome measures and assessment schedule.

		Assessment Schedule					
Measure	Measure Description	Baseline	Daily	1m-5m (30, 60, 90, 120, 150d)	6m (180d)	+3m (270d) F/U	+6m (360d) F/U
Demographic Information:	Self-reported descriptive data (age, gender, race, ethnicity, education, marital status, work status, COVID history, care partner data, care recipient data, etc.)	X					
Care Recipient Medical Record Information	Medical record data for the person with TBI (time since diagnosis, injury severity, imaging findings, etc.)	X					
Caregiver Appraisal Scale (CAS) [53]	Care partner-reported evaluation of the positive and negative aspects of caregiving (47 items); existing support for reliability & validity [54, 55]	X					
Mayo-Portland Adaptability Inventory- Fourth Edition (MPAI-4) [56]	Proxy-report measure designed to evaluates functional ability for persons with TBI (35 items); existing support for reliability & validity [57–59]	X					
Posttraumatic Stress Disorder Checklist for DSM-5 (PCL-5) [60]	Evaluates symptoms of posttraumatic stress disorder (PTSD) defined by the Diagnostic and Statistical Manual of Mental Disorders, 5^th^ edition (20 items); Adapted for proxy assessment of symptoms in person with TBI (care partner is the proxy); existing support for reliability and validity [60–73]	X					
Supervision Rating Scale (SRS) [74]	Care partner rates the amount of “supervision” that the person with TBI receives on a single item; existing support for reliability & validity [74]	X					
TBI-CareQOL Caregiver Strain SF [34, 35]	Evaluates self-reported feelings of caregiver burden; existing support of reliability & validity [33–35]	X		X	X	X	X
TBI-CareQOL Caregiver-Specific Anxiety SF [34, 36]	Evaluates self-reported feelings of worry and anxiety about the person with TBI; existing support for reliability & validity [33, 34, 36]	X		X	X	X	X
PROMIS Sleep-Related Impairment SF [75]	Assesses self-reported impact of poor sleep on daytime functioning; existing support for reliability and validity [34, 75–80]	X		X	X	X	X
PROMIS Fatigue SF [76, 81]	Assesses self-reported fatigue and the impact this fatigue has on functioning; existing support for reliability and validity [34, 76–79, 82–88]	X		X	X	X	X
PROMIS Anxiety SF [76, 81]	Evaluates self-reported anxiety and worry; existing support for reliability, validity, & responsiveness [34, 77–79, 87, 89]	X		X	X	X	X
PROMIS Depression SF [76, 81]	Evaluates self-reported feelings of depression or sadness; existing support for reliability, validity, & responsiveness [34, 77–79, 87, 89, 90]	X		X	X	X	X
PROMIS Anger SF [76, 81]	Evaluates self-reported feelings of anger and frustration; existing support for reliability, validity, & responsiveness [34, 77, 79, 89]	X		X	X	X	X
NIH Toolbox Self-Efficacy-General SF [91]	Evaluates self-reported efficacy or confidence in one’s ability to engage in day to day tasks; existing support for reliability, validity, & responsiveness [91–93]	X		X	X	X	X
Neuro-QoL Positive Affect & Well-Being SF [94]	Evaluates self-reported positive outlook and perceptions of overall well-being; existing support for reliability, validity, & responsiveness [94]	X		X	X	X	X
NIH Toolbox Perceived Stress [91]	Evaluates self-reported coping with everyday events; existing support for reliability validity, & responsiveness [91]	X		X	X	X	X
PROMIS Ability to Participate in Social Roles & Activities SF [76, 81]	Evaluates self-reported perceptions about one’s ability to engage in social activities; existing support for reliability, validity & responsiveness [34, 77, 79, 87, 95]	X		X	X	X	X
PROMIS Global Health V1.2	Evaluates self-reported perceptions of overall health and well-being, including physical, mental, and social health (10 items); existing support for reliability, validity & responsiveness [96–100]	X		X	X	X	X
COVID HRQOL	Self-reported rating of concern or worry about COVID-19 (single item rating that ranges from 0 to 10)	X		X	X	X	X
Single-item Caregiver Strain [34, 35]	Evaluates self-reported feelings of burden related to the caregiver role		X				
Single-item Anxiety [76, 81]	Evaluates self-reported feelings of worry and anxiety		X				
Single-item Depression [76, 81]	Evaluates self-reported feelings of sadness and depression		X				
Fitbit^®^-based estimate of physical activity	Includes step count data and data about intensity of physical activity		X				
Fitbit^®^-based estimate of sleep	Includes sleep duration and duration of time for different sleep stages		X				
Medical History /Medications/ Treatments/ COVID Questionnaire	Self-reported medical history and treatment data	X		3m (90d)	X	X	X
AE/Status Update	Self-reported adverse events			3m (90d)	X	X	X
Feasibility	Evaluates self-reported perceptions about the overall experience within the study				X		

We will examine change scores from baseline to 6-months in self-reported caregiver strain as measured by TBI-CareQOL Caregiver Strain. Secondary and tertiary objectives include the examination of efficacy of the JITAI to improve other HRQOL outcomes (including other aspects of self-reported physical, mental and social health, as well as Fitbit^®^-based estimates of physical activity and sleep). Again, we will examine change scores from baseline to 6-months for both the self-reported HRQOL measures, as well as the Fitbit^®^ -based estimates of physical activity and sleep. We hypothesize that the care partners who receive the intervention (JITAI group) will show improvements in Caregiver Strain (Primary endpoint) and other aspects of HRQOL (as measured by the other self-report measures) and functioning (as measured by the Fitbit^®^ -based estimates of physical activity and sleep) after the 6-month (180 day) home monitoring period. Tertiary analyses will include the examination of change over time in HRQOL and estimates of functioning for other time frames (e.g., change over time from month to month, maintenance of changes at 3- and 6-months post-intervention, etc.). We expect that the care partners who receive the intervention (JITAI group) will show improvements in HRQOL for these shorter timeframes, and we anticipate that these gains will be maintained at 3- and 6-months post-intervention. Tertiary analyses will also include the identification of care partners at the greatest risk for negative physical and mental HRQOL outcomes, and the times when they are most at risk. For these analyses we hypothesize that objective, data-derived mobile phenotypes can predict risk for adverse HRQOL (as measured by the TBI-CareQOL item banks) in these care partners.

### Data collection, storage, and protection

This project uses multiple electronic data capture and management platforms (e.g., REDCap, CareQOL, Qualtrics, Fitbit^®^, University of Michigan Health Information Technology and Services server, Google Cloud, Amazon Web Services Cloud). All platforms are designed for human subjects research and comply with federal and local data and information security practices. Each site will use the same platforms. Annual data audits will be conducted at each data collection site and will include the review of at least 20% of the cases that have been collected since the previous review.

### Sample size considerations

Initial power calculations were based on the expected difference in change in self-reported TBI-CareQOL Caregiver Strain (from baseline to 6-month follow-up) between the JITAI group and the control group. Using self-reported TBI-CareQOL Caregiver Strain, we expect using a normative T-score, with a standardized mean set at 50 and a standard deviation of 9.66, the minimum detectable difference for caregiver strain to be in the range of 4–6 points. A sample size of 92 in each group will have 80% power to detect a difference in means of 4.0 assuming that the common standard deviation is 9.66 using a two-group t-test with a 0.05 two-sided significance level. Similar results would be expected for the expected difference in change in the other HRQOL measures that are the focus of the secondary and tertiary objectives.

The proposed sample size of n = 240 caregivers accounts for sampling estimates for the tertiary analyses that consider multiple HRQOL outcomes simultaneously. For these analyses, sample size estimates were based on the following considerations: [37] 1) type I error rate (α) = 0.05; 2) the smallest meaningful difference to be detected = (δ); 3) power (γ) = 0.8; 4) an assumed outcome marginal variance (σ^2^) that is constant over time and 10^2^ (according to the T-metric used to score each HRQOL domain); 5) the number of repeated measurements per person (n) = 1 baseline, 8 post-baseline for a total of n = 9 repeated assessments; and 6) an assumed exchangeable correlation (ρ) = a range of values 0.4, 0.5, 0.6, 0.7, 0.8 for the correlation structure among the repeated measurements. The sample size formula is:

$$\text{N}=\frac{4{\left\{Z_{1-\frac{\alpha }{2}}+Z_{1-\gamma }\right\}}^{2}\sigma_{C}^{2}}{\delta^{2}},$$

where

$$\sigma_{C}^{2}=Var\left(C_{i}\right)\left[1-{\left\{Corr\left(Y_{i0},\;C_{i}\right)\right\}}^{2}\right]=\;\frac{\left(1-\rho \right)\left\{1+\left(n-1\right)\rho \right\}\sigma^{2}}{n-1}.$$

Table 2 provides the sample sizes needed for different effect sizes (δ) and correlations (ρ) when comparing post-baseline averaged outcomes between JITAI and control groups using an analysis of covariance method (assumed 15% attrition rate [11, 38, 39]). Thus, the proposed sample size of N = 240 care partners is sufficient to detect small, but meaningful effects.

**Table 2:** Estimated sample sizes.

Effect sizes (T-metric)	Correlation (ρ)				
	*0*.*40*	*0*.*50*	*0*.*60*	*0*.*70*	*0*.*80*
*5*	234	230	198	146	82
*10*	60	58	50	38	22
*15*	26	26	22	16	10

### Statistics

Care partners in each study group (JITAI and control) will be compared descriptively according to Consolidated Standards of Reporting Trials Guidelines [40]. T-tests/analysis of variance will be used to examine group differences for continuous variables (e.g., age, HRQOL outcomes, Fitbit^®^ outcomes, time since injury, functional status of person with TBI). Chi-squared/Fisher exact tests will be used to examine group differences for categorical variables (e.g., sex, ethnicity, race, education, marital status, relationship to person with TBI, TBI severity).

As mentioned above, we will examine change scores from baseline to 6-months in self-reported caregiver strain as measured by TBI-CareQOL Caregiver Strain (Primary Endpoint), as well as other secondary and tertiary HRQOL and functional outcome endpoints. For these analyses, we will examine score changes from baseline to 6-months post-intervention separately for each of these outcome measures between the two arms. Means and standard deviations of the within-person changes will be calculated. In addition, separate models will be conducted for each dependent variable (Caregiver Strain or other HRQOL or functional outcome). Each model will use a linear mixed effect model, where a random intercept will be included to account for repeated measurements from the same subject. In addition, the following independent variables will be included in each model: the randomization arm, time and interaction between randomization arm and time, and baseline Caregiver Strain/HRQOL/Functional outcome scores. Key biologic variables such as age, sex, TBI severity, and co-morbidities will be among some of the factors explored as potential confounders. Differences between treatment arms can then be obtained by estimating and testing the corresponding parameters in the linear mixed effect model.

Tertiary analyses will also include comparing the averaged post-baseline outcomes (physical activity, sleep and HRQOL) between the JITAI and control groups, as well as the monthly rate of change in outcomes (physical activity, sleep and HRQOL) between the JITAI and control groups. Assuming one pre-specified TBI-CareQOL HRQOL domain as the primary outcome variable, we will compare the averaged post-baseline outcomes between the JITAI and control groups (one baseline assessment, six end-of-month assessments, and two post-intervention assessments). We will adjust for baseline measurements to improve the power of the two-sample t-test to determine whether mean post-baseline HRQOL differs between the two groups. We will compare monthly rate of change using models that consider both time-invariant and time-varying covariates. Generalized linear mixed models will also be performed to complement the results of the t-test and adjust for specific baseline care partner/person with TBI characteristics (e.g., demographic, TBI severity) to increase the precision of our inference. Finally, tertiary analyses will also include kernel methods in machine learning to estimate interpretable “signatures” preceding episodes of adverse symptoms or decreases in TBI-CareQOL HRQOL scores in at least one domain that have potential to inform timely intervention [41]. We will make inferences about which Fitbit^®^ mobile-sensor-collected behavioral phenotypes are most predictive and evaluate their time-delayed impact upon TBI-CareQOL HRQOL scores at different time lags.

### Ethics and dissemination

This trial is being carried out in accordance with the United States (US) Code of Federal Regulations (CFR) applicable to clinical studies (45 CFR Part 46, 21 CFR Part 50, 21 CFR Part 56, 21 CFR Part 312, and/or 21 CFR Part 812) and research best practices. The protocol, informed consent document, and all participant materials have received approval from IRBMED (9/18/2020), which is serving as the institutional review board (IRB) of record for both data collection sites (IRBMED Multi-site Application Approval HUM00181282; IRBMED University of Michigan Site Application Approval HUM00186921; IRBMED Baylor College of Medicine Site Application Approval SITE0000087; Baylor College of Medicine/Memorial Hermann IRB number H-48478. This trial is also registered with ClinicalTrials.gov (NCT04570930).

All study results will be reported in accordance with CONSORT 2010 guidelines and the 2013 CONSORT-PRO extension guidance [42, 43]. In addition, this minimal risk study is being monitored by an Independent Safety Monitor (ISM). While we do not anticipate any serious adverse events for this trial, the ISM is responsible for evaluating trial progress (including data quality and timeliness, recruitment, accrual and retention, participant risk versus benefit, overall performance of the trial and other factors that can affect outcomes) in order to ensure the safety of study participants.

## Results

Recruitment to this trial began in November 2020; data collection is expected to take ~4 years (November 2024), and the dissemination of trial results is planned thereafter. We expect results for the primary outcomes in the winter of 2025.

## Discussion

This protocol provides a description of the design and methods for a randomized clinical trial that is designed to examine the efficacy of a self-management JITAI. JITAIs use real-time data collection to inform and personalize the delivery of the intervention and provide an accessible and cost-effective approach that delivers personalized and adaptive interventions in a real-time, real-world context [44, 45]. This emerging approach been associated with significant improvements in health outcomes in behavioral health, including physical activity, [46, 47] alcohol use, [48, 49] mental illness, [50] and smoking cessation [51, 52].

This study will be the first time a self-management JITAI, or indeed any JITAI, will be examined in caregivers of persons with TBI. This is especially important given interventions that focus on the self-care of care partners are critically important to improving health outcomes for both care partners and persons with TBI. Care partners may recognize the importance of self-care; however, the time demands of caring for an individual with TBI, as well as the associated physical and emotional toll, make it difficult for these individuals to prioritize their own self-care. In fact, care partners of persons with TBI often indicate that they simply forget to engage in health management strategies focused on their own well-being [6]. Self-management programs (i.e., programs focused on medical/behavioral management, role management, and emotional management [7]) provide a potential avenue for fostering care partner self-care, yet only three studies have been published examining a self-management approach in care partners of persons with TBI (two of which involved several in-person treatment sessions and the other which involved the completion of educational modules, as well as 8–10 supportive phone calls [one call delivered every 2-weeks]) [7–11].

This JITAI is administered by the *CareQOL app* (usable with Android and iOS platforms), which integrates passive sensor data derived from a Fitbit^®^ (e.g., accelerometer-based estimates of physical activity and sleep), alongside real-time self-reported ratings of HRQOL (that are administered as a computer adaptive test over the course of the week). This app was modeled after the one that was developed and tested in our previous work examining the efficacy of a similar JITAI in medical interns [31, 32].

## Conclusions

At the conclusion of this project, we will have the data needed to support a low-cost, low-burden self-management JITAI in care partners of persons with TBI and will better understand how HRQOL may fluctuate on a day-to-day basis in these individuals. We will also be able to identify care partners of persons with TBI at greatest risk for negative physical and mental health outcomes. Ultimately, this work may result in a scalable intervention that can be widely implemented in this population, leading to improved HRQOL for care partners of persons with TBI and those they care for. Importantly, the findings from this study may be able to be adapted and applied to other caregiving groups, further amplifying the impact of this work.

## Supporting information

S1 Checklist (DOC)

S1 Protocol (DOCX)

## Abbreviations

HRQOL: Health-related quality of life
IRB: Institutional review board
ISM: Independent Safety Monitor
JITAI: Just in time adaptive intervention
RCT: Randomized control trial
TBI: Traumatic Brain Injury
