# Supplementary material for: Improving outcomes for care partners of persons with traumatic brain injury: Protocol for a randomized control trial of a just-in-time-adaptive self-management intervention
Source: PLoS One. 2022 Jun 9;17(6):e0268726. doi: 10.1371/journal.pone.0268726 (PMC9182304; doi:10.1371/journal.pone.0268726)
Supplement: S1 Protocol — (DOCX) [file pone.0268726.s002.docx]

**Improving Outcomes for Care Partners of Persons with Traumatic Brain Injury**

**Protocol Number: HUM000181282**

**National Clinical Trial (NCT) Identified Number: NCT04570930**

**Principal Investigator:** **Noelle E. Carlozzi, Ph.D.**

**Sponsor: University of Michigan**

**Grant Title: Improving Outcomes for Care Partners of Persons with Traumatic Brain Injury**

**Grant Number: 2R01NR013658**

**Funded by: National Institute of Nursing Research, National Institutes of Health**

**Version Number: v.3.0**

**21 April 2022**

**Protocol Amendment History:**

| **Version** | **Date** | **Description of Change** | **Brief Rationale** |
| --- | --- | --- | --- |
| 2.0 | 2/9/2021 | Added clinicaltrials.gov number on cover page and updated version date and number and table of contents page numbers. | Administrative update |
| 2.0 | 2/9/2021 | In sections 8.1, 8.1.5.3 and 8.1.5.4, noted that the monthly and follow-up surveys could also be given in an interview format (in addition to the CareQOL app). | Provides flexibility with administration |
| 3.0 | 4/21/2022 | Updated version date and number and table of contents page numbers. | Administrative update |
| 3.0 | 4/21/2022 | Deleted confidentiality statement. | Protocol will not be confidential (will be published and posted on clinicaltrials.gov). |
| 3.0 | 4/21/2022 | In sections 1.1, 1.2 and 4.1, the number of participants was increased from 240 to up to 280. BCM participants were increased from 120 to 160. | Enrollment was increased to allow BCM to enroll more participants and to replace participants who dropped out of the study before the end of the 6-month home monitoring period. |
|  |  |  |  |
|  |  |  |  |
|  |  |  |  |
|  |  |  |  |
|  |  |  |  |
|  |  |  |  |
|  |  |  |  |
|  |  |  |  |
|  |  |  |  |
|  |  |  |  |
|  |  |  |  |
|  |  |  |  |
|  |  |  |  |

Table of Contents

[STATEMENT OF COMPLIANCE 1](#_Toc63779691)

[INVESTIGATOR’S SIGNATURE 2](#_Toc63779692)

[1 PROTOCOL SUMMARY 3](#_Toc63779693)

[1.1 Synopsis 3](#_Toc63779694)

[1.2 Schema 5](#_Toc63779695)

[1.3 Schedule of Activities 6](#_Toc63779696)

[2 INTRODUCTION 6](#_Toc63779697)

[2.1 Study Rationale 6](#_Toc63779698)

[2.2 Background 7](#_Toc63779699)

[2.3 Risk/Benefit Assessment 8](#_Toc63779700)

[2.3.1 Known Potential Risks 8](#_Toc63779701)

[2.3.2 Known Potential Benefits 10](#_Toc63779702)

[2.3.3 Assessment of Potential Risks and Benefits 10](#_Toc63779703)

[3 OBJECTIVES AND ENDPOINTS 10](#_Toc63779704)

[4 STUDY DESIGN 11](#_Toc63779705)

[4.1 Overall Design 11](#_Toc63779706)

[4.2 End-of-Study Definition 11](#_Toc63779707)

[5 STUDY POPULATION 12](#_Toc63779708)

[5.1 Inclusion Criteria 12](#_Toc63779709)

[5.2 Exclusion Criteria 12](#_Toc63779710)

[5.3 Lifestyle Considerations 13](#_Toc63779711)

[5.4 Screen Failures 13](#_Toc63779712)

[5.5 Strategies for Recruitment and Retention 13](#_Toc63779713)

[6 STUDY INTERVENTION(S) OR EXPERIMENTAL MANIPULATION(S) 14](#_Toc63779714)

[6.1 Study Intervention(s) or Experimental Manipulation(s) Administration 14](#_Toc63779715)

[6.2 Fidelity 15](#_Toc63779716)

[6.3 Concomitant Therapy 16](#_Toc63779717)

[7 STUDY INTERVENTION/EXPERIMENTAL MANIPULATION DISCONTINUATION AND PARTICIPANT DISCONTINUATION/WITHDRAWAL 17](#_Toc63779718)

[7.1 Participant Discontinuation/Withdrawal from the Study 17](#_Toc63779719)

[7.2 Lost to Follow-Up 17](#_Toc63779720)

[8 STUDY ASSESSMENTS AND PROCEDURES 17](#_Toc63779721)

[8.1 Endpoint and Other Non-Safety Assessments 18](#_Toc63779722)

[8.2 Adverse Events and Serious Adverse Events 23](#_Toc63779723)

[8.2.1 Definition of Adverse Events 23](#_Toc63779724)

[8.2.2 Definition of Serious Adverse Events 23](#_Toc63779725)

[8.2.3 Classification of an Adverse Event 23](#_Toc63779726)

[8.2.4 Time Period and Frequency for Event Assessment and Follow-Up 24](#_Toc63779727)

[8.2.5 MULTI-SITE Adverse Event Reporting 24](#_Toc63779728)

[9 STATISTICAL CONSIDERATIONS 25](#_Toc63779729)

[9.1 Statistical Hypotheses 25](#_Toc63779730)

[9.2 Sample Size Determination 25](#_Toc63779731)

[9.3 Populations for Analyses 26](#_Toc63779732)

[9.4 Statistical Analyses 27](#_Toc63779733)

[9.4.1 General Approach 27](#_Toc63779734)

[9.4.2 Analysis of the Primary Endpoint(s) 27](#_Toc63779735)

[9.4.3 Analysis of the Secondary Endpoint(s) 27](#_Toc63779736)

[9.4.4 Baseline Descriptive Statistics 28](#_Toc63779737)

[10 SUPPORTING DOCUMENTATION AND OPERATIONAL CONSIDERATIONS 28](#_Toc63779738)

[10.1 Regulatory, Ethical, and Study Oversight Considerations 28](#_Toc63779739)

[10.1.1 Informed Consent Process 28](#_Toc63779740)

[10.1.2 Confidentiality and Privacy 29](#_Toc63779741)

[10.1.3 Future Use of Stored Specimens and Data 30](#_Toc63779742)

[10.1.4 Key Roles and Study Governance 31](#_Toc63779743)

[10.1.5 Safety Oversight 31](#_Toc63779744)

[10.1.6 Quality Assurance and Quality Control 32](#_Toc63779745)

[10.1.7 Data Handling and Record Keeping 33](#_Toc63779746)

[10.1.8 OTHER REPORTABLE INCIDENTS & OCCURRENCES (ORIOS) 34](#_Toc63779747)

[10.1.9 Publication and Data Sharing Policy 35](#_Toc63779748)

[11 REFERENCES 37](#_Toc63779749)

[12 APPENDIX 1: MULTI-SITE RESEARCH REPORTING PLAN 40](#_Toc63779750)

# STATEMENT OF COMPLIANCE

This trial will be carried out in accordance with the United States (US) Code of Federal Regulations (CFR) applicable to clinical studies (45 CFR Part 46, 21 CFR Part 50, 21 CFR Part 56, 21 CFR Part 312, and/or 21 CFR Part 812) and research best practices. The PI and all study team members who are responsible for the conduct, management, or oversight of NIH-funded clinical trials will complete Human Subjects Protection and best practices training.

The protocol, informed consent document, and all participant materials will be submitted to IRBMED, which is serving as the IRB of record for both sites, for review and approval. Approval of both the protocol and the consent documents will be obtained before any participant is consented. Any amendment to the protocol will be submitted for review and approval by IRBMED before the changes are implemented to the study. All changes to the consent form(s) will be IRB approved; a determination will be made regarding whether a new consent needs to be obtained from participants who provided consent, using a previously approved consent form.

# INVESTIGATOR’S SIGNATURE

The signature below constitutes the approval of this protocol and provides the necessary assurances that this study will be conducted according to all stipulations of the protocol, including all statements regarding confidentiality, and according to local legal and regulatory requirements and applicable US federal regulations and research best practices.

Principal Investigator and Clinical Site Investigator:

| Signed: |  | Date: |  |
| --- | --- | --- | --- |
|  | Name: Noelle E. Carlozzi, Ph.D. | | |
|  | Title: Associate Professor  Affiliation: Department of Physical Medicine & Rehabilitation, University of Michigan  Address: 2800 Plymouth Rd., NCRC Bldg. 14, Ann Arbor, MI 48109-2800  Telephone: 734-763-8917  Email: carlozzi@med.umich.edu | | |

Clinical Site Investigator:

| Signed: |  | Date: |  |
| --- | --- | --- | --- |
|  | Name: Angelle Sander, Ph.D. | | |
|  | Title: Associate Professor  Affiliation: Baylor College of Medicine  Address: 7200 Cambridge, Ste. 10C, Houston, TX 77030  Telephone: 713-797-7161  Email: [asander@bcm.edu](mailto:asander@bcm.edu) | | |

# PROTOCOL SUMMARY

## Synopsis

**Title:** Improving Outcomes for Care Partners of Persons with Traumatic Brain Injury

**Grant Number:** 2R01NR013658

**Study Description:** Informal family care partners of persons with traumatic brain injury (TBI) are often faced with considerable physical and emotional stress resulting from their caregiver role. Not surprisingly, this stress is associated with depression, anxiety, fatigue, isolation, sleep disturbance, and physical health problems for the care partner. This is especially problematic since these problems also inadvertently affect health and well-being (i.e., outcomes) of the person with TBI. Given this, there is a need for interventions to improve health-related quality of life (HRQOL) for these care partners. Yet, the time demands of caring for an individual with a TBI, as well as the associated physical and emotional toll, make it difficult for care partners to prioritize their own self-care. Therefore, it is critical that interventions are low burden, and easy to engage in, or else there is little hope that the intervention will improve care partner HRQOL.

This study proposes using the TBI-CareQOL, a newly developed HRQOL measurement system specific to care partners of TBI, to evaluate the effectiveness of a personalized health management behavioral intervention aimed at improving the HRQOL of care partners. This personalized intervention, called the just-in-time adaptive intervention (JITAI), uses physical activity and sleep data collected using a wrist-worn device (i.e., a Fitbit®) and real-time self-reports of HRQOL (caregiver strain, anxiety and depression) collected via a mobile app, to provide personalized feedback via app alert.

Participants in this study will be randomized either to a control group, where they will wear the Fitbit® and provide daily reports of HRQOL over a six-month (180 day) period (without the personalized feedback), or the JITAI group, where they will wear the Fitbit®, provide daily reports of HRQOL and receive personalized pushes for 6 months. Up to 280 participants will be enrolled; 140 in the control group and 140 in the JITAI group.

We hypothesize that the care partners who receive the intervention (JITAI group) will show improvements in caregiver strain and mental health (depression and anxiety) after the 6-month (180 day) home monitoring period.

**Objectives:**

**Primary Objective:** Examine the efficacy of the JITAI to improve self-reported caregiver strain in care partners of persons with traumatic brain injury (TBI).

**Secondary Objectives:** Examine the efficacy of the JITAI to improve self-reported mental health (depression and anxiety) in care partners of persons with traumatic brain injury (TBI).

**Endpoints:**

**Primary Endpoint:** Change from baseline in self-reported caregiver strain as measured by TBI-CareQOL Caregiver Strain at the end of the 6-month (180 day) intervention period.

**Secondary Endpoints:** 1) Change from baseline in self-reported depression as measured by PROMIS Depression at the end of the 6-month (180 day) intervention period; and 2) Change from baseline in self-reported anxiety as measured by PROMIS Anxiety at the end of the 6-month (180 day) intervention period.

**Study Population:** Up to 280 care partners of persons with TBI will participate in this study. Participant recruitment and enrollment will take place at two data collection sites – the University of Michigan and Baylor College of Medicine. Care partners must be at least 18 years old, be able to read and understand English and be caring for an adult (age 18 or above) with a medically documented TBI who is at least 1 year post-injury who sustained their TBI at age 16 or older. They must be willing to use their personal mobile device (e.g., smartphone, tablet) for this study, be willing to download the study app (CareQOL) and Fitbit® app and be willing to complete all study assessments.

**Phase or Stage:** Not applicable; behavioral intervention.

**Description of Sites Enrolling Participants:** There are two data collection sites for this study: The University of Michigan in Ann Arbor, MI (n=120 care partners of persons with TBI) and Baylor College of Medicine in Houston, TX (n=160 care partners of persons with TBI).

**Description of Study Intervention:** Just-in-time adaptive intervention (JITAI) is a mobile health behavior-change approach that operationalizes the selection and delivery of personalized mobile device intervention strategies based on real-time data collection. In this study, a study-specific app (CareQOL app) will integrate sensor data from a Fitbit® (on physical activity and sleep) with real-time self-report ratings of HRQOL (caregiver strain, depression, anxiety) to inform the JITAI. The JITAI will deliver personalized messages via the app ~50% the days during the intervention period.

**Study Duration:** Data collection is expected to take 4 years.

**Participant Duration:** The participant duration in the study is ~375 days (a little over 1 year).

## Schema

## Schedule of Activities

|  | Pre-enrollment | Enrollment  Day -10 | ~ 12 months^1^ | | | | | | | | |
| --- | --- | --- | --- | --- | --- | --- | --- | --- | --- | --- | --- |
|  |  |  | Run-in^2^  Days -10 to -1 | End of months 1-5 assessments  30, 60, 90, 120, 150 (+/- 7 days) | | | | | End of 6 month assessment^3^  180 (+/- 7 days) | +3m F/U Assessment  270 (+/- 30 days) | +6m F/U Assessment  360 (+/- 30 days) |
| TBI Documentation | X |  |  |  |  |  |  |  |  |  |  |
| Caregiver Eligibility | X | X |  |  |  |  |  |  |  |  |  |
| Informed Consent |  | X |  |  |  |  |  |  |  |  |  |
| Demographics/Baseline Survey |  | X |  |  |  |  |  |  |  |  |  |
| Caregiver Appraisal Scale |  | X |  |  |  |  |  |  |  |  |  |
| MPAI-4 |  | X |  |  |  |  |  |  |  |  |  |
| PCL-5 |  | X |  |  |  |  |  |  |  |  |  |
| SRS |  | X |  |  |  |  |  |  |  |  |  |
| TBI Medical Record Confirmation CRF |  | X |  |  |  |  |  |  |  |  |  |
| HRQOL Outcome Measures |  | X |  | X | X | X | X | X | X | X | X |
| Fitbit® & CareQOL app Instructions |  | X |  |  |  |  |  |  |  |  |  |
| Randomization |  | X |  |  |  |  |  |  |  |  |  |
| JITIA^4,5^ or Control Home Monitoring^5^ |  |  |  | | | | | | |  |  |
| Daily EMA^6^ |  |  |  | | | | | | |  |  |
| Feasibility/acceptability questionnaire |  |  |  |  |  |  |  |  | X |  |  |
| Medications/Therapies/Med History/COVID |  | X |  |  |  | x |  |  | X | X | X |
| Adverse Events Reporting |  |  |  |  |  | x |  |  | X | X | X |
| 1 – Individual participant duration may vary depending on when they complete their HRQOL assessments; 2 – Approximately 10 days in duration, to include time for shipping and at least 3-4 days of data collection; 3 – Primary outcome assessment time point; 4 – Active intervention, includes personalized push notifications; 5 – Includes daily wearing the Fitbit® for sleep and physical activity monitoring for both groups; 6 – for both active and control groups | | | | | | | | | | | |

# INTRODUCTION

## Study Rationale

**SIGNIFICANCE**

Illness impacts the entire family, and the complete picture of human disease is a collage of the experiences of both the affected patient and family care partner(s).^1^ As a society, responsibility for addressing these needs has always been placed on family care partners, who face an enormous and growing burden providing care to a loved one while trying to maintain their own health and well-being (e.g., HRQOL).^2^

Traumatic brain injury (TBI) is a particularly challenging condition for family care partners due to the unexpected burden of providing prolonged supportive care at home once the survivor has been discharged from the hospital or rehabilitation facility. Care partners of persons with TBI must not only learn to cope with dramatic changes in their loved one’s health and functional abilities, but they also may feel ill-equipped to provide the requisite level of care.

Care partners of persons with TBI commonly experience problems with physical and mental HRQOL as a result of the caregiver role. While interventions exist to help improve these care partners’ HRQOL, they are typically time-intensive and expensive, and have limited success at improving HRQOL in these individuals. Despite clear advantages in terms of convenience, reach and scalability with using mobile technologies (including JITAIs) to support healthy behavior change, their clinical utility in care partners of persons with TBI remains untested. We will address this critical gap in the literature by using passive data (i.e., physical activity and sleep data derived from the Fitbit®) and real-time assessments of HRQOL to identify and provide support to care partners of persons with TBI at the greatest risk for negative outcomes. We will also assess the effectiveness of a low-cost, low-burden intervention (the JITAI) designed to improve HRQOL outcomes in these care partners.

## Background

**Preliminary Studies (Care Partners of Persons with TBI).**

Under the leadership of Noelle Carlozzi (PI), the first funding period of this R01 (NINR/NIH R01NR013658 (09/27/12 – 06/30/18) resulted in the publication of over 27 papers.^3-28^ In addition, data have been presented to institutional, national, international, and community audiences (29 presentations have been conducted to date). The overall purpose of the initial R01 was to identify important HRQOL domains that are relevant for care partners of persons with TBI (Aim 1), as well as to develop (Aim 2) and validate (Aim 3) a PRO measurement system that captures both the generic and more unique aspects of HRQOL in care partners of persons with TBI, with the goal of being able to use this measurement system to inform the development and assess the efficacy of clinical interventions designed to improve care partner HRQOL. Findings indicated the pervasive negative impact that caring for an individual with a TBI can have across mental, physical and social HRQOL; the new measurement system, the TBI-CareQOL,^7^ includes 24 different measures of HRQOL and is now ready to be used in clinical trials designed to improve care partner HRQOL.

**Preliminary Studies (JITAI Feasibility and Efficacy).**

The proposed JITAI study methodology is modeled after and informed by the current findings from the Intern Health Study (R01MH101459; PI Srijan Sen). To date, the Intern Health study includes three separate cohorts. In the first cohort, physical activity and sleep were assessed using Fitbit® trackers, and daily ratings of mood were completed via text for N=38 medical interns. In this study, 92% of participants provided sleep, activity, and mood data on at least 80% of days. Data showed an expected strong association between sleep and mood, but found that sleep predicted mood the following day substantially more strongly than mood predicted subsequent sleep (Sleep🡪Mood b=0.12; p<0.001; Mood🡪Sleep b=0.05; p=0.04; **Figure**). They also found that, on a given night, the farther an individual’s sleep midpoint was from their pre-internship baseline midpoint, the lower their mood (p<0.001).^29^ Most recently, Sen and colleagues extended this work to a larger randomized controlled trial comparing a JITAI with usual care (n=2053). This trial employed microrandomization (which involved repeatedly randomizing each individual to receive various intervention feedback in order to determine the efficacy of different classes and types of mobile data feedback, and assess the moderating influence of location and timing on the effectiveness of the feedback). Findings from this work indicated that the optimal study design involved participants receiving feedback on ~50% of the days during the six-month intervention period (thus we have elected to implement this feedback strategy in this protocol). Further, they found that receptivity to mobile health interventions was moderated by participants’ current state related to the target of the intervention. For instance, interventions around increasing sleep duration were most effective when participants had low sleep the night before, adding further conceptual backing for the JITAI approach. Study findings also indicated that medical interns who were randomized to the JITAI intervention demonstrated significant improvements in depression.


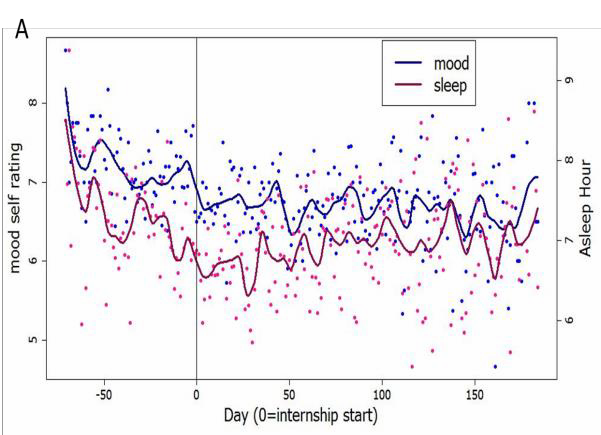


**Figure**

## Risk/Benefit Assessment

### Known Potential Risks

The risks associated with the proposed study are minor and infrequent and include risks to: i) confidentiality; ii) frustration, stress and/or anxiety; iii) and skin irritation:

1. Confidentiality. Confidentiality is a concern in this study. Every possible effort will be made to keep the research information in the strictest confidence, but we cannot absolutely guarantee that accidental disclosure will not happen. We remind participants that the responsibility of confidentiality rests with everyone: they should think carefully before discussing their role in this study with anyone since the effects of disclosure are unknown. Additionally, during the 6-month home monitoring period, participants may receive alerts when the participant is not in a private area. Participants will be informed that it is up to them where they complete the daily assessments, and if it is inconvenient/uncomfortable for them to complete the assessments when prompted they can wait and do it when it is more convenient/private or skip that assessment.

To reduce the risk to confidentiality, no personal identifying information will appear on or with the participant data, where possible. Each participant will be assigned a participant ID by the study team to maintain confidentiality. The participant’s self-report data and CRF data will be identified with the participant ID. Additionally, an access code will be assigned to each participant which they will use to enroll in the CareQOL app. Participants will register their study Fitbit® on the Fitbit® app using a username and email address of their choice. The study team will help the participant configure the Fitbit® app for minimal sharing; however, participants may opt to change those settings on their own (without study team knowledge). The data collected on the Fitbit® will be linked to the CareQOL app by user authentication token, which is comprised of a random series of characters that is used to securely query for user activity data; no identifying information (including email address or Fitbit® user ID) is saved in the CareQOL app.

Each site will keep a master list of their site’s participant names and matching participant IDs. This master list will not be shared with the other site, and will be kept by site personnel in a password-protected file on a secure server or in a secure environment (e.g., locked cabinet, restricted access). No one other than the study team at the study site will have access to their site’s master list (except when needed for study monitoring). Informed consents will be stored in a study-specific database which contains only the informed consents and the informed consents for each site will only be available to their site’s study team members (except when needed for study monitoring). The electronic data collection and storage systems used (e.g., CareQOL app, REDCap, Qualtrics, Fitbit®) are secure, web-based systems, with access to the study data limited to authorized individuals.

Additionally, participants in this study are covered under a NIH Certificate of Confidentiality (CoC). The CoC limits the disclosure of identifiable, sensitive information to a few instances: 1) if required by Federal, State, or local laws (e.g., as required by the Federal Food, Drug, and Cosmetic Act, or state laws requiring the reporting of communicable diseases to State and local health departments), excluding instances of disclosure in any Federal, State, or local civil, criminal, administrative, legislative, or other proceeding; 2) if necessary for the medical treatment of the individual to whom the information, document, or biospecimen pertains and made with the consent of such individual; 3) if made with the consent of the individual to whom the information, document, or biospecimen pertains; or 4) if made for the purposes of other scientific research that is in compliance with applicable Federal regulations governing the protection of human subjects in research. Participants will be notified in the informed consent that this study is covered by a CoC and will be informed of the protections and limitations to protections provided by the CoC. Lastly, the identities of all participants will be held in strict confidence to the extent provided by law. If findings from the study are published and/or presented at a professional meeting, no participant will be identified by name.

1. Fatigue, Frustration, Stress and/or Anxiety. It is possible that participants may experience fatigue, frustration or feel inconvenienced when completing the self-report surveys, when receiving the personalized interventions from the CareQOL app or from wearing the Fitbit®. Participants may also feel fatigue, stress and/or anxiety related to completing the study activities. Participants will be instructed that they can skip any questions that make them feel uncomfortable, and if they receive an app alert when it isn’t convenient for them, they can wait and open the app when it is more convenient.
2. Skin Irritation. Some participants may get skin irritation from wearing the Fitbit®. Participants will be instructed to remove the device if this occurs and to contact the study team. Their band may be swapped out for a different type of wristband if they experience irritation. We offer a wide variety of wristbands to help make the Fitbit® as comfortable as possible.

To minimize the potential for risks, participants will be briefed in detail as to what they will experience throughout the study. All participants are informed that they may terminate participation from the study at any time without penalty.

### Known Potential Benefits

The proposed study aims to investigate the effectiveness of the JITAI to promote mental and physical health self-management in care partners of persons with TBI, as well as assess care partners at the greatest risk of negative mental and physical HRQOL outcomes. While there may not be any direct benefits to participants, it is possible that participants may find that the JITAI improves their mood, stress levels, and/or overall quality of life. Additionally, some participants may also have a positive benefit from wearing the Fitbit®, as it may make them more aware of their activity levels and sleep. Overall, we expect that the findings from this study will help researchers further develop a personalized, easy to deploy, clinical intervention for care partners of persons with TBI.

### Assessment of Potential Risks and Benefits

As described above, there are minimal risks for the study participants and several potential benefits. Thus, the risk/benefit ratio is highly favorable.

# OBJECTIVES AND ENDPOINTS

| **Objectives** | **Endpoints** |
| --- | --- |
| Primary | |
| Examine the efficacy of the JITAI to improve self-reported caregiver strain in care partners of persons with traumatic brain injury (TBI) | Change from baseline in self-reported caregiver strain as measured by TBI-CareQOL Caregiver Strain at the end of the 6-month (180 day) intervention period |
| Secondary | |
| Examine the efficacy of the JITAI to improve self-reported mental health (depression and anxiety) in care partners of persons with traumatic brain injury (TBI) | Change from baseline in self-reported depression as measured by PROMIS Depression at the end of the 6-month (180 day) intervention period |
|  | Change from baseline in self-reported anxiety as measured by PROMIS Anxiety at the end of the 6-month (180 day) intervention period |
| Tertiary/Exploratory/Other | |
| All other objectives and endpoints (e.g. sleep and physical activity variables, other mental and physical health domains, etc.) are considered tertiary | |

# STUDY DESIGN

## Overall Design

**Study Design:** This behavioral trial will use a two-arm randomized controlled design. Each of the up to 280 care partner participants will be randomized to an active “JITAI” arm or to a control arm. The random allocation of participants to the treatment arm or control arm establishes the basis for testing the statistical significance or difference between the groups in HRQOL (caregiver strain, depression and anxiety).

**Randomization:** Blocked randomization will be used to limit bias and achieve an equal distribution of participants to the control and treatment arms. A randomization list will be generated for each site and the study statistician will oversee randomization. The participant will be randomized once he/she is deemed eligible and has provided informed consent (i.e. at Enrollment prior to baseline data collection). The study coordinator/research assistant who consented the participant will use their site’s randomization list to assign the participant to the correct study arm.

**Duration of Study Participation:** Study participation for both arms of the study (control and JITAI) involves a baseline assessment, followed by an approximate 10-day run-in period then a 6-month (180 day) home monitoring period (in which the intervention will be administered to the JITAI group). After the completion of the 6-month (180 day) home monitoring period, post-intervention surveys will be administered at 3 months (90 days) and 6 months (180 days) after the end of the 6-month (180 day) home monitoring period. Thus, the total duration of the study is ~375 days.

**Study Sites**: This is a multi-site study involving two sites: University of Michigan and Baylor College of Medicine. University of Michigan is the lead site and their IRBMED will serve as the single IRB for this study.

**Study Intervention:** The just in time adaptive intervention (JITAI) uses sensor data derived from the Fitbit® (e.g., accelerometer-based estimates of physical activity and sleep) and real-time self-report ratings (assessed once daily) of HRQOL (caregiver strain, depression, anxiety) to deliver personalized “pushes” to participants via a study specific app (CareQOL app). The personalized pushes will be delivered on ~50% of the days over the 6-month intervention period.

## End-of-Study Definition

While the primary outcome assessment is at the end of the 6-month (180 day) home monitoring period, the end of the study is defined as completion of the final follow-up survey administered 6 months (180 days) after end of the home monitoring period shown in the Schedule of Activities (SoA), **Section 1.3**.]

# STUDY POPULATION

## Inclusion Criteria

In order to be eligible to participate in this study, an individual must meet all of the following criteria:

1. Provide informed consent
2. Be at least 18 years of age
3. Be able to read and understand English
4. Be caring for an adult (age 18 or above) with a medically documented TBI that is ≥1-year post-injury and meets the TBI Model Systems (TBIMS) criteria* for complicated mild, moderate or severe TBI and who sustained their TBI at age 16 or older
5. Provide emotional, physical, and/or financial support/assistance to the individual with the TBI, indicating a response ≥1 to the following question: “On a scale of 0-10, where 0 is “no assistance” and 10 is “assistance with all activities”, how much assistance does the person you care for require from you to complete activities of daily living due to problems resulting from his/her TBI? Activities could consist of personal hygiene, dressing and undressing, housework, taking medications, managing money, running errands, shopping for groceries or clothing, transportation, meal preparation and cleanup, remembering things, etc.”
6. Have access to necessary resources for participating in a technology-based intervention (smartphone/tablet and internet access) and be willing to use their personal equipment/internet for this study, including downloading the study app and the Fitbit® app on their mobile device
7. Is able and willing to complete all study assessments for the duration of their study participation (approximately 375 days)

*TBIMS Criteria: TBI is defined as having had damage to brain tissue caused by external mechanical force. Complicated mild TBI is defined as an emergency room GCS score of 13-15 with positive findings on neuroimaging. Moderate to severe TBI is defined by at least one of the following: 1) post traumatic amnesia greater than 24 hours, 2) trauma related intracranial neuroimaging abnormalities, 3) loss of consciousness greater than 30 minutes not due to sedation or intoxication, 4) Glasgow Coma Scale in the emergency room of less than 13, not due to intubation, sedation or intoxication.

## Exclusion Criteria

An individual who meets the following criteria will be excluded from participation in this study:

1. Is a professional, paid caregiver (e.g., home health aide)
2. Anything that would preclude safe or meaningful participation in the study

## Lifestyle Considerations

During this study, participants are asked to:

- Wear a Fitbit® 24 hours/day, except when charging during the 10-day run-in period and 6-month (180 day) home monitoring period.
- Install the CareQOL app and Fitbit® app on their personal mobile device, and keep these apps installed for the duration of the study (~375 days).

## Screen Failures

Screen failures are defined as participants who consent to participate in this study but are not subsequently assigned to the study intervention or entered in the study. Screen failures are unlikely in this study because eligibility assessment occurs pre-informed consent. However, if during the 10-day run-in period before the 6-month home monitoring period starts the study team is made aware of something that would change their eligibility, the participant will be noted as a screen failure.

## Strategies for Recruitment and Retention

Recruitment: Two sites (University of Michigan [U-M] – Ann Arbor, MI; and Baylor College of Medicine [BCM] - Houston, TX) will be used to recruit participants (care partners of persons with TBI) for this study. Sites will recruit participants primarily through their established medical and TBI clinics, participant registries (both TBI- and care partner-specific registries), and clinical databases. Study participants will also be recruited through local TBI and care partner support groups, from organizations like the Brain Injury Association, and through targeted Facebook ads.

Recruitment and retention methods used in this study will be approved by the Institutional Review Board at U-M (U-M IRBMED; serving as the single IRB for this study for BCM and U-M) and are detailed in the IRBMED submission. Potential participants will be recruited directly or through the person they are caring for. Individuals who are interested in participating will be encouraged to ask questions about the study and their participation, and if they opt to enroll, they will provide informed consent prior to completing any study assessments.

In order to ensure that recruitment targets are being met, teleconference calls with the study recruitment teams by the project manager will be conducted to review progress, discuss problems, and guide additional recruitment efforts to meet stratification goals. Stratification goals (e.g., race, ethnicity, gender, etc.) can be found in the manual of procedures for this study.

Retention: Given the intensive nature of this study, we expect that participants will miss some daily assessments and have times when they do not wear the Fitbit®; our retention plan is focused on minimizing these missed assessments and engaging participants throughout the study.

Retention will be facilitated by several strategies:

- App development will consider factors known to increase participant engagement including strategies to target engagement and minimize fatigue (easy to navigate platform, app tailoring based on Fitbit® sensor feedback, etc.).^30^ The app will also include a study team dashboard that we will use to monitor participant engagement.
- User guides and contact information for the study team (phone and email) will be supplied to participants and they will be encouraged to contact the study team with any questions or concerns.
- When enrolling participants, we will offer participants a choice of wristbands; they can select the wristband that is most comfortable and visually appealing to them. If they are not happy with their selection, we will provide an alternate wristband.
- Study staff will maintain regular contact with the study participants. All study participants will be contacted at least once during the study run-in period, at least once during the first week of the 6-month home monitoring period and at least once each month during the remaining months of the 6-month home monitoring period.
- Participants who do not complete any study assessments (e.g., uploading Fitbit® data or answering EMAs) for several days will receive a reminder from the study team.
- In case of lost equipment/equipment failure during the run-in and home monitoring periods, we will send replacements as supplies allow.

Participant Incentives: We will compensate participants for their time and inconvenience, as follows:

- - Baseline assessment, final 6-month home monitoring assessment, 3-month post-monitoring assessment, 6-month post-home monitoring assessment: Participants will receive $20 for completing each survey ($80 total possible).
  - Monthly HRQOL surveys during the home monitoring period: Participants will complete end of month surveys during the home monitoring period. Participants will receive $10 for completing each end of month survey for months 1-5 ($50 total possible).
  - Daily EMA assessments and Fitbit® data during the 6-month home monitoring period: Participants will receive $1 per day for each day that they have EMA and/or Fitbit® data during the six-month (180 day) home monitoring period ($180 total possible).
  - Participants who complete the study will be allowed to keep the Fitbit® that they use in the study.
- Compensation will occur monthly to encourage prompt responding.
- We may send out nominal value items (e.g., notepads, pens) throughout the duration of study participation to encourage engagement.

# STUDY INTERVENTION(S) OR EXPERIMENTAL MANIPULATION(S)

## Study Intervention(s) or Experimental Manipulation(s) Administration

All participants will receive a Fitbit ® for the collection of sleep and activity (steps) data, and will download the Fitbit® app and study app, CareQOL, on their personal mobile device (iOS or Android). The CareQOL app will deliver ecological momentary assessments (EMAs) once per day, compile and display data (including that collected on the Fitbit®), and deliver study notifications, including messages for the participant to complete the daily EMA and other study surveys as well as delivering the personalized study intervention prompts to the intervention group (Just-in-time adaptive intervention [JITAI] group).

All study participants will complete 3 EMA questions daily on the CareQOL app. Each participant will be prompted by a push notification in a five-hour window (based on participant preference) from the app to answer the questions. The EMA questions are comprised of: 1 question on caregiver strain (taken from the TBI-CareQOL Caregiver Strain item bank), 1 question on anxiety (taken from the PROMIS Anxiety item bank) and 1 question on depression (taken from the PROMIS Depression item bank). Questions are on a five-point scale, with higher scores indicating more of the named construct.

In addition to the collection of the EMA data, the app complies and displays a graphical summary of historical data for caregiver stress (strain), worry (anxiety), sadness (depression), steps (collected by the Fitbit®) and hours of sleep (collected by the Fitbit®) on a participant dashboard. Participants can view this information for the past week, past month or past year. This is available to all participants as a pull – that is, it is available at all times but is accessed only if the user chooses to access it.

In this study, half of the participants will be randomized to receive the intervention (JITAI; described below); the remaining participants will be in the control group, who will not receive the JITAI but will complete the activities already described in this section. Participants who are randomized to receive the JITAI will have a 50/50 chance of receiving the intervention each day.

The JITAI aims to promote behavioral change through motivational messages that are delivered through the CareQOL app as push notifications. These notifications provide a trigger for participants to initiate or continue behavior change and/or monitor behaviors (through engagement with the with the app). The push notifications are low burden: participants can personalize the administration time (in a 5-hour window) and notifications can be viewed quickly on their phone’s lock screen. Participants can also choose not to engage with the notification at the time it is sent if it is inconvenient – they can return to it later if needed.

The JITAI push notifications are aimed at promoting healthy behaviors (physical activity and good sleep hygiene) and improving mood (anxiety, depression, caregiver strain). If receiving a notification, the message will be randomly drawn from this pool of messages. Some messages will use participants’ data directly in the messages (e.g., You walked an average of 8,120 steps this week), and most of messages will be personalized based on data (e.g., someone with low steps will get a different message than someone with medium steps than someone with high steps; high-medium-low). Messages are comprised of one or more of the following different types: 1) Data feedback; 2) Facts; 3) Tips; and 4) Support. Randomization of the days the participants receive messages and the message the participant receives from the pool will be done through the CareQOL app.

The study intervention period is 180 days (6 months); in addition, there is an ~10-day run-in period where participants will install/adjust/troubleshoot the study technology and the app will gather baseline data to use to tailor messages once the intervention period begins.

## Fidelity

For this study, the intervention will be administered via the CareQOL app. The study team will conduct training during the baseline visit with each participant which will include:

- Helping the participant download and register for the CareQOL and Fitbit® apps on their personal device
- Helping the participant set any relevant app settings (e.g., make sure alerts are turned on/off, privacy settings, etc.)
- Demonstrating the CareQOL app features to the participant (e.g., participant dashboard, etc.)
- Ensuring that the participant understands the importance of entering the daily ratings and wearing the Fitbit®
- Explaining the relationship between the daily ratings and Fitbit® data and the intervention prompts (JITAI group only)
- Demonstrating how to sync the Fitbit® (including using the Fitbit® app) and how to enter the daily ratings in the CareQOL app

During the 10-day run-in period between the baseline visit and the start of the intervention, study team members will assist participants to make sure that participants can:

- Successfully wear and sync the Fitbit®
- Successfully respond to the EMA prompts

Participants who are unable to successfully complete the run-in period will be terminated from the study and noted as a screen failure.

Training and training materials will be provided to all study team members. New study members will undergo training before the study is started. Training will be documented in the electronic regulatory binder.

Participant adherence to the protocol will be assessed throughout the study. Study staff will have access to the CareQOL app dashboard which can be used to monitor participant engagement and adherence with the app. Specifically, study staff will monitor:

- Adherence to EMA entry
- Adherence to Fitbit® use/syncing
- Adherence to completing the end of month surveys (~30, 60, 90, 120, 150, 180 day)
- Adherence to completing the 3-month (~270 days) and 6-month (~360 days) post-intervention surveys

Adherence will be calculated as a percentage for each of these areas for all participants who are not withdrawn or lost to follow-up.

## Concomitant Therapy

There are no concomitant treatment or management strategy restrictions for this study. Information about current treatments (medications and non-medications) will be collected at baseline. Changes in treatments will be captured at the end of month 3 (~90 days) and month 6 (~180 days) during the home monitoring period, and 3-months (~270 days) and 6-months (~360 days) post-home monitoring period.

# STUDY INTERVENTION/EXPERIMENTAL MANIPULATION DISCONTINUATION AND PARTICIPANT DISCONTINUATION/WITHDRAWAL

## Participant Discontinuation/Withdrawal from the Study

Participants are free to withdraw from participation in the study at any time upon request.

An investigator may discontinue a participant from the study for the following reasons:

- Lost-to-follow up; unable to contact participant (see **Section 7.2, Lost to Follow-Up**)
- Any event or medical condition/situation that occurs that would indicate that continued collection of follow-up study data would not be in the best interest of the participant or might require treatment that would confound the interpretation of the study
- The participant meets an exclusion criterion (either newly developed or not previously recognized) that precludes further study participation
- The participant is non-compliant with the study protocol

The reason for participant discontinuation or withdrawal from the study will be recorded. Participants who sign the informed consent form and are randomized but do not complete the 10-day run-in period will be replaced. Participants who withdraw or are discontinued before the end of the 6-month (~180 day) home monitoring period will be replaced.

## Lost to Follow-Up

A participant will be considered lost to follow-up if: 1) he or she does not complete the end of month survey at the end of the 6-month (~180 day) home monitoring period ***and*** 2) study staff are unable to contact the participant after a minimum of 3 attempts.

The following actions must be taken if a participant fails to complete the end of the month survey at the end of the 6-month (~180 day) intervention period:

- The site will attempt to contact the participant and counsel the participant on the importance of completing the survey and ascertain if the participant wishes to and/or should continue in the study
- The site will make every effort to regain contact with the participant (where possible, 3 telephone calls/emails/texts and, if necessary, a certified letter to the participant’s last known mailing address or local equivalent methods) before a participant is deemed lost to follow-up. These contact attempts will be documented in the participant’s study file.
- Participants that continue to be unreachable will be considered to have withdrawn from the study (with a primary reason recorded as lost to follow-up).

# STUDY ASSESSMENTS AND PROCEDURES

## Endpoint and Other Non-Safety Assessments

Participants will complete a baseline assessment, daily and monthly assessments during a 6-month (~180 day) home monitoring period, and two follow-up assessments. Study data will be collected via self-report into web or app-based surveys and a consumer wearable (Fitbit®).

Eligibility and baseline procedures and assessments can be conducted either virtually via teleconference/webinar or in-person. Daily, monthly and follow-up assessments will be conducted virtually through the CareQOL app; monthly or follow-up assessments may also be conducted by interview.

**8.1.1 TBI DOCUMENTATION**

We will verify and document TBI status and injury severity of the care recipient for all participants. Data must conform to the TBI Model Systems classification, and be obtained from official medical documentation (e.g. medical records).

**8.1.2 CARE PARTNER ELIGIBILITY**

Confirmation of care partner eligibility according to criteria listed in Section 5, above, will be documented on the Eligibility CRF. It will occur prior to informed consent being obtained and before any study procedures associated with baseline assessment. Eligible individuals will then participate in the consent process, followed by randomization and baseline data collection.

**8.1.3 DEMOGRAPHICS & CHARACTERIZATION**

All participants will complete a series of surveys designed to characterize the sample with respect to demographics, caregiving experiences, and usual coping or management strategies.

- Participant Demographics. A study-designed form will be used to capture demographic data including age, gender, race, ethnicity, education, marital status, work status, COVID history/status, care partner data, care recipient data, etc. Administration time for this assessment is ~5-10 minutes.
- Medical History, Medications, Treatments, COVID Questionnaire. Study designed forms will be used to capture medical history and current treatments/management strategies (medication and non-medication – e.g., exercise, mindfulness) and COVID history. Administration time for this assessment is 10 minutes.
- Caregiver Appraisal Scale (CAS). The CAS assesses positive and negative aspects of the caregiving role. Four separate subdomain scores (perceived burden, caregiver relationship satisfaction, caregiving ideology, and caregiving mastery) can be calculated; higher scores indicating better functioning. Administration time for this 47-item measure is ~5-10 minutes.
- Mayo-Portland Adaptability Inventory- Fourth Edition (MPAI-4). The MPAI-4 is a 35-item proxy-report measure designed to assess functional ability for persons with TBI. Higher scores indicate poorer functional ability. The administration time for this measure is ~5-10 minutes.
- Posttraumatic Stress Disorder Checklist for DSM-5 (PCL-5). This version of the PCL-5 has been adapted for the proxy (in this study, the care partner is the proxy) assessment of posttraumatic stress disorder (PTSD) symptoms in the person with TBI. The PCL-5 is a 20-item measure that assesses the 20 symptoms of PTSD defined by the Diagnostic and Statistical Manual of Mental Disorders, 5^th^ edition (DSM–5). A total symptom severity score, as well as 5 cluster scores can be obtained from this measure; higher scores indicate more PTSD symptomatology. Administration time for this 20-item measure is ~5-10 minutes.
- Supervision Rating Scale (SRS). This is a single rating that the caregiver provides about the overall amount of “supervision” that the person with TBI receives. Ratings range from 1-13; higher ratings indicate greater levels of required supervision. Administration time is ~2 minutes.
- Care Recipient Medical Record Information. Study staff will complete a CRF with information about the person with TBI for whom the care partner is providing care (e.g., time since diagnosis, details of diagnosis, disease stage/severity, imaging findings etc.). This data will be entered into the study database by study staff.

**8.1.4 FITBIT® AND CAREQOL APP**

Participants will be taught how to install both the Fitbit® and CareQOL apps onto their personal mobile devices. Participants will also to be taught how to create and login to the necessary accounts. See section 6.2 for details regarding participant education.

- The study team will provide an access code for login to the CareQOL app.
- Participants will use a user id and personal email address of their choosing for their Fitbit® account.
- Data collected from the Fitbit® will be securely transmitted to the CareQOL app by user authentication token, which is comprised of a random series of characters that is used to securely query for user activity data.
- Participants will view their Fitbit® sleep and step-count data through the CareQOL app, though there are not restrictions on them also viewing the information in the Fitbit® app.

**8.1.4.1 FITBIT®**

All participants will don a wrist-worn Fitbit® with heart rate recording capabilities for the duration of the 10-day run-in period and 6-month (180 day) home monitoring period to monitor activity and sleep. While we intend to use the Fitbit Inspire HR®, we reserve the option to adopt a comparable device in the event of supply limitations or changes to the product line as long as there is minimal impact on the intervention delivery or primary outcome data integrity.

- Participants may use a personal Fitbit® if it has comparable features.
- Participants are expected to wear the Fitbit® continuously except for when charging and uploading data.
- Participants are expected to sync the Fitbit® daily.

**8.1.4.2 CAREQOL APP**

The study app (CareQOL) is the participant-facing platform for all daily, monthly and follow-up assessments, for viewing sleep and step count data from the Fitbit®, and for intervention delivery. After the home monitoring period is over (e.g. after the 6-month [180 day] assessment), the app will go into an “assessment only” mode during which only the +3 month (270 day) and +6-month (360 day) follow-up assessments will be activated. The daily and monthly assessments, the link with the Fitbit®, and intervention delivery will cease after the home monitoring period has elapsed.

**8.1.4.3 RUN-IN HOME MONITORING PERIOD**

A 10-day run-in period will follow the baseline assessment. This period is to allow shipping time of the Fitbit® and provide the participant time to familiarize themselves with the study technology (Fitbit®, CareQOL app) and procedures. This period will also allow the study team to troubleshoot any potential barriers or issues that arise before the official start of the 6-month (180 day) home monitoring period. It also allows for data collection that can be used to inform the intervention messages once the home monitoring period begins.

**8.1.5 HRQOL ASSSESSMENT**

Participants will complete a battery of HRQOL surveys; some will be administered daily and be used to drive the intervention, and others will be administered at baseline, monthly and during follow-up. The individual survey administration schedule is shown in the Table, and descriptions are provided below.

**Table: HRQOL Assessment Schedule**

|  | **Baseline** | **Daily*** | **1m-5m (30, 60, 90, 120, 150d)** | **6m (180d)** | **+3m (270d) F/U** | **+6m**  **(360d) F/U** |
| --- | --- | --- | --- | --- | --- | --- |
| Caregiver Strain SF | X |  | X | X | X | X |
| Caregiver-Specific Anxiety SF | X |  | X | X | X | X |
| Sleep-Related Impairment SF | X |  | X | X | X | X |
| Fatigue SF | X |  | X | X | X | X |
| Anxiety SF | X |  | X | X | X | X |
| Depression SF | X |  | X | X | X | X |
| Anger SF | X |  | X | X | X | X |
| Self-Efficacy SF | X |  | X | X | X | X |
| Positive Affect & Well-Being SF | X |  | X | X | X | X |
| Perceived Stress | X |  | X | X | X | X |
| Ability to Participate in Social Roles & Activities SF | X |  | X | X | X | X |
| Global Health | X |  | X | X | X | X |
| COVID HRQOL | X |  | X | X | X | X |
| Single-item Caregiver Strain |  | X |  |  |  |  |
| Single-item Anxiety |  | X |  |  |  |  |
| Single-item Depression |  | X |  |  |  |  |
| MedHistory/Medications/Treatment/COVID | X |  | 3m (90d) | X | X | X |
| AE/Status Update |  |  | 3m (90d) | X | X | X |
| Feasibility |  |  |  | X |  |  |
| *Daily surveys will be administered through the Run-in and 6-month home monitoring periods. | | | | | | |

- TBI-CareQOL Caregiver Strain Short Form (SF). TBI-CareQOL Caregiver Strain assesses perceived feelings of feeling overwhelmed, stressed and “beat-down” related to the care partner role. This measure is scored on a *T* metric (M = 50; SD = 10). Higher scores indicate more strain. The administration time for this measure is ~1 minute.
- TBI-CareQOL Caregiver-Specific Anxiety SF. TBI-CareQOL Caregiver-Specific Anxiety assesses perceived feelings of worry and anxiety specific to the safety, health, and future well-being of the person with TBI. This measure is scored on a *T* metric (M = 50; SD = 10). Higher scores indicate more anxiety. The administration time for this measure is ~1 minute.
- PROMIS Sleep-Related Impairment SF. PROMIS Sleep-Related Impairment evaluates the effect of poor sleep on daytime functioning. This measure is scored on a *T* metric (M = 50; SD = 10). Higher scores indicate more sleep-related impairment. Administration time for this measure is ~1 minute.
- PROMIS Fatigue SF. PROMIS Fatigue evaluates self-reported symptoms of fatigue, ranging from mild subjective feelings of tiredness to overwhelming exhaustion that may decrease one’s ability to perform activities of daily living. This measure is scored on a *T* metric (M = 50; SD = 10). Higher scores indicate more fatigue. Administration time for this measure is ~1 minute.
- PROMIS Anxiety SF. PROMIS Anxiety assesses self-reported feelings of fear, anxiety and hyperarousal. This measure is scored on a *T* metric (M = 50; SD = 10). Higher scores indicate more anxiety. Administration time for this measure is ~1 minute.
- PROMIS Depression SF. PROMIS Depression assesses self-reported feelings of sadness and worthlessness. This measure is scored on a *T* metric (M = 50; SD = 10). Higher scores indicate more depression. Administration time for this measure is ~1 minute.
- PROMIS Anger SF. PROMIS Anger assesses self-reported feelings of irritability and frustration. This measure is scored on a *T* metric (M = 50; SD = 10). Higher scores indicate more anger. Administration time for this measure is ~1 minute.
- NIH Toolbox Self-Efficacy. NIH Toolbox Self-Efficacy assesses self-reported confidence in the ability to successfully perform specific tasks or behaviors related to one’s overall functioning. This measure is scored on a *T* metric (M = 50; SD = 10). Higher scores indicate more self-efficacy. Administration time for this measure is ~1 minute.
- Neuro-QoL Positive Affect & Well-Being SF. Neuro-QoL Positive Affect and Well-Being assesses parts of an individual’s life that are related to overall life meaning and purpose, well-being and satisfaction. This measure is scored on a *T* metric (M = 50; SD = 10). Higher scores indicate greater satisfaction. Administration time is ~1 minute.
- NIH Toolbox Perceived Stress SF. NIH Toolbox Perceived Stress is a self-report measure designed to assess an individual’s feelings about the nature of events and individual coping resources. This measure is scored on a *T* metric (M = 50; SD = 10). Higher scores indicate more perceived stress. Administration time is ~1 minute.
- PROMIS Ability to Participate in Social Roles and Activities SF. PROMIS Ability to Participate in Social Roles and Activities assesses involvement in one’s ability to participate in usual social roles and activities. This measure is scored on a *T* metric (M = 50; SD = 10). Higher scores indicate more ability to participate. Administration time is ~1 minute.
- PROMIS Global Health v1.2. This 10-item patient-reported outcome measure assesses overall physical, mental, and social health. This measure is scored on a *T* metric (M = 50; SD = 10); separate scores are generated for physical and mental health. Higher scores indicate better health. The administration time for this measure is ~3 minutes.
- COVID HRQOL. This 1-item patient-reported outcome measure asks how concerned the participant is about COVID-19 from 0 – 10. The administration time for this measure is less than 1 minute.

**8.1.5.1 BASELINE ASSESSMENTS**

Participants will be prompted by the CareQOL after registration to complete the baseline HRQOL assessments. Administration time is 10-15 minutes.

**8.1.5.2 DAILY ASSESSMENTS**

Participants will be prompted by the CareQOL app to complete a brief 3-item assessment once per day during the run-in and six-month (180 day) home monitoring periods. These questions will be answered directly on the app and involve <1 minute administration time in total. The daily assessments will include:

- TBI-CareQOL Caregiver Strain – single-item assessment to evaluate self-reported caregiver strain. The individual items that are administered each day will vary.
- PROMIS Anxiety – single item assessment to evaluate self-reported anxiety. The individual items that are administered each day will vary.
- PROMIS Depression – single item assessment to evaluate self-reported depression. The individual items that are administered each day will vary.

**8.1.5.3 MONTHLY ASSESSMENTS**

Participants will be prompted by the CareQOL app to complete a series of self-report surveys at the end of each month (day 30, 60, 90, 120, 150, 180) during the 6-month (180 day) home monitoring period. They will also complete a post-intervention/app feasibility survey at the end of month 6 (day 180). These surveys will be answered directly on the CareQOL app and/or by interview, and should take ~10-15 minutes to complete.

- Study Feasibility: At the 6-month (180 day) assessment only, participants will complete a Study Feasibility survey to assess the experience of the participant with the study methodology and technology, including the CareQOL app, Fitbit®, and the JITAI. Administration time is 5 minutes.

Additionally, participants will update their medical history, medications, therapies, and COVID history and be queried about AEs at the end of month 3 (day 90) and month 6 (day 180).

- Medical History, Medications, Treatments, COVID Questionnaire. Participants will update their medical history and current treatments/management strategies (medication and non-medication – e.g., exercise, mindfulness) and COVID history. Administration time for this assessment is 10 minutes
- Adverse Events. Participants will be asked about any changes in mental or physical health. Administration time is 5 minutes.

**8.1.5.4 FOLLOW-UP ASSESSMENTS**

Participants will be prompted by the CareQOL app to complete a series of self-report surveys at +3-months (270 days) and +6-months (360 days) follow-up. As with the monthly surveys, responses will be directly entered into the CareQOL app and/or by interview and should take ~10-15 minutes to complete. Additionally, participants will update their medical history, medications, treatments and COVID history and be queried about AEs at these time points.

## Adverse Events and Serious Adverse Events

### Definition of Adverse Events

The U-M IRBMED definition of an adverse event will be used for this study. The definition can be found at <https://az.research.umich.edu/medschool/glossary> and is below:

An adverse event (AE) is any experience or abnormal finding that has taken place during the course of a research project and was harmful to the subject participating in the research, or increased the risks of harm from the research, or had an unfavorable impact on the risk/benefit ratio. The event may or may not be caused by an intervention. Adverse events also include psychological, social, emotional or financial harms.

### Definition of Serious Adverse Events

The U-M IRBMED definition of a serious adverse event (SAE) will be used for this study. The definition can be found at <https://az.research.umich.edu/medschool/glossary> and is below:

A serious adverse event (SAE) is any adverse experience occurring at any dose or level of participation that results in any of the following outcomes:

- Results in death,
- Is life-threatening,
- Requires inpatient hospitalization or prolongation of existing hospitalization,
- Results in persistent or significant disability/incapacity, or
- Is a congenital anomaly/birth defect.

### Classification of an Adverse Event

#### Severity of Event

All AEs will be assessed by the principal investigator, and/or if necessary, a study co-investigator or the study’s independent safety monitor.

The following guidelines will be used to describe severity.

- **Mild** – Events require minimal or no treatment and do not interfere with the participant’s daily activities.
- **Moderate** – Events result in a low level of inconvenience or concern with the therapeutic measures. Moderate events may cause some interference with functioning.
- **Severe** – Events interrupt a participant’s usual daily activity and may require systemic drug therapy or other treatment. Severe events are usually potentially life-threatening or incapacitating. Of note, the term “severe” does not necessarily equate to “serious”.

#### Relationship to Study INTERVENTION/Experimental Manipulation

All adverse events (AEs) will have their relationship to study procedures, including the intervention, assessed by an appropriately-trained investigator based on temporal relationship and his/her clinical judgment. All AEs will be categorized according to the likelihood that they are related or not related to the study intervention.

- **Related (Possible, Probable, Definite)**
  - The event is a known or suspected effect of the intervention or research procedures (e.g., listed in the protocol documents including, consent, publications, etc.)
  - There is at least a reasonable temporal relationship between the intervention or procedure and the event onset
  - The event abates when the intervention is discontinued
  - The event reappears upon a re-challenge with the intervention
  - The event includes data that was collected solely for research purposes
  - The event included disturbing or upsetting questions asked solely for research purposes
- **Not Related (Unlikely, Not Related)**
  - The event is NOT a known or suspected effect of the study intervention or procedures
  - The event is readily explained by characteristics of the study population
  - There is no temporal relationship between the intervention and event onset
  - An alternate etiology has been established

#### Expectedness

An investigator with appropriate expertise will be responsible for determining whether an adverse event (AE) is expected or unexpected. An event that is expected has been addressed or described in one or more of the following: Informed consent document for this study, IRB application for this study, grant application or study agreement, protocol or procedures for this study, ISR Reports, published literature, other documentation, or characteristics of a study population. An AE will be considered unexpected if the nature, severity, or frequency of the event is not consistent with the risk information described.

### Time Period and Frequency for Event Assessment and Follow-Up

The occurrence of an AE or SAE may come to the attention of the study team via spontaneous report or formal assessment, where participants will be asked to describe any physical or mental health worries/concerns since the last check-in (see SOA for time points). Regardless of the reporting mechanism, the study team will probe any concerns to get additional information and determine if it is an adverse event. Details will be documented in the participant study record.

Any side effects that are determined to be adverse events related to study participation will be documented on the study adverse event form in the study REDCap database and followed until resolution.

### MULTI-SITE Adverse Event Reporting

For this study, only related adverse events will be reported. Adverse events will be reported to U-M IRBMED and NINR using the standard IRBMED reporting schedule: *(*<https://az.research.umich.edu/medschool/guidance/adverse-event-reporting>). The U-M IRBMED reporting schedule is dependent on the severity of the event, and whether such adverse events were expected. Any serious adverse events will be reported to the ISM, U-M IRBMED and NINR as soon as possible but not later than 7 days of learning of the event. Non-threatening potentially serious adverse events that are causally related to the research will be reported within 14 days of learning of the event to the ISM, U-M IRBMED and NINR.

Sites will report study-related adverse events using the adverse event form in the REDCap study database promptly. In the case of a serious adverse event, the project PI and project manager should be notified by email or phone as soon as possible but not later than 7 days of learning of the event. Adverse events will be discussed at investigator and project staff meetings.

# STATISTICAL CONSIDERATIONS

## Statistical Hypotheses

Primary Endpoint:

1) We hypothesize greater decreases in caregiver strain will be observed among participants in the JITAI group (relative to the control group) at the end of the 6-month home monitoring period.

Secondary Endpoints:

1) We hypothesize greater decreases in anxiety will be observed among participants in the JITAI group (relative to the control group) at the end of the 6-month home monitoring period.

2) We hypothesize greater decreases in depression will be observed among participants in the JITAI group (relative to the control group) at the end of the 6-month home monitoring period.

## Sample Size Determination

Our initial power calculations were based on the expected difference in change in self-reported TBI-CareQOL Caregiver Strain (from baseline to 6-month follow-up) between the JITAI intervention group and the control group. Using self-reported TBI-CareQOL Caregiver Strain, we expect using a normative T-score, with a standardized mean set at 50 and a standard deviation of 9.66, the minimum detectable difference (MID) for caregiver strain to be in the range of 4-6 points. A sample size of 92 in each group will have 80% power to detect a difference in means of 4.0 assuming that the common standard deviation is 9.66 using a two group t-test with a 0.05 two-sided significance level.

Similar results would be expected for the expected difference in change in self-reported PROMIS Depression (from baseline to 6-month follow-up) between the JITAI intervention group and the control group. Using self-reported PROMIS Depression, we expect using a normative T-score, with a standardized mean set at 52.03 and a standard deviation of 9.79, the minimum detectable difference (MID) for depression to be in the range of 4-6 points. A sample size of 95 in each group will have 80% power to detect a difference in means of 4.0 assuming that the common standard deviation is 9.79 using a two group t-test with a 0.05 two-sided significance level.

Similar results would also be expected for the expected difference in change in self-reported PROMIS Anxiety (from baseline to 6-month follow-up) between the JITAI intervention group and the control group. Using self-reported PROMIS Anxiety, we expect using a normative T-score, with a standardized mean set at 54.8 and a standard deviation of 9.53, the minimum detectable difference (MID) for depression to be in the range of 4-6 points. A sample size of 90 in each group will have 80% power to detect a difference in means of 4.0 assuming that the common standard deviation is 9.53 using a two group t-test with a 0.05 two-sided significance level.

With regard to the exploratory analyses, sample size estimates used the following inputs:^31^ 1) type I error rate ($\alpha$) = 0.05; 2) the smallest meaningful difference to be detected = ($\delta$); 3) power ($\gamma$) = 0.8; 4) we assumed outcome marginal variance ($\sigma^{2}$) is constant over time and ${10}^{2}$ (according to the T-metric used to score each HRQOL domain); 5) the number of repeated measurements per person (n) = 1 baseline, 8 post-baseline for a total of n=9 repeated assessments; and 6) with regard to the correlation structure among the repeated measurements we assumed an exchangeable correlation ($\rho$) = a range of values 0.4, 0.5, 0.6, 0.7, 0.8. The sample size formula is:

$N=\frac{{4\left\{ Z_{1-\frac{\alpha}{2}}+Z_{1-\gamma} \right\}}^{2}\sigma_{C}^{2}}{\delta^{2}}$, where $\sigma_{C}^{2}=Var\left( C_{i} \right)\left[ 1-\left\{ Corr\left( Y_{i0}, C_{i} \right) \right\}^{2} \right]= \frac{\left( 1-\rho\right)\left\{ 1+\left( n-1 \right)\rho\right\}\sigma^{2}}{n-1}$

| **Table. Estimated Sample Sizes** | | | | | |
| --- | --- | --- | --- | --- | --- |
| Effect sizes  (T-metric) | Correlation ($\rho$) | | | | |
|  | *0.40* | *0.50* | *0.60* | *0.70* | *0.80* |
| *5* | 234 | 230 | 198 | 146 | 82 |
| *10* | 60 | 58 | 50 | 38 | 22 |
| *15* | 26 | 26 | 22 | 16 | 10 |

The **Table** provides the sample sizes needed for different effect sizes ($\delta$) and correlations ($\rho$) when comparing post-baseline averaged outcomes between JITAI and control groups using an ANCOVA method (assumed 15% attrition rate^32-34^). Thus, the proposed sample size of N=240 care partners is sufficient to detect small, but meaningful effects.

Thus, the proposed sample size comfortably exceeds those required to address the primary and secondary endpoint analyses, and is sufficient to examine the tertiary and exploratory endpoints.

## Populations for Analyses

The primary and secondary endpoints will compare the JITAI group with the control group. Tertiary analyses may also compare important subgroups (e.g., caregiver groups that differ by relationship type [parent vs. spousal caregivers], caregiver groups that differ by sex [male vs. female caregivers], caregiver groups that differ according to the functional status of the person with TBI (e.g., complicated mild vs. moderate vs. severe TBI or perceived functional status based on Mayo Portland Adaptability Index scores).

The analyses will be an intention-to-treat approach where the participant will contribute data to the arm he/she was randomized to regardless of the amount of data contributed (i.e. the duration of participation).

## Statistical Analyses

### General Approach

Continuous measures will be described using means and standard deviations (or medians and ranges if non-normality is detected), while categorical measures will be summarized with frequencies and percentages.

For inferential tests, a two group t-test with a 0.05 two-sided significance level will be used.

Analyses for primary, secondary, and tertiary endpoints are described individually, below.

The distribution of the data will be examined to ensure that parametric testing is appropriate. In cases where data distributions are not normal, nonparametric tests will be used to analyze the data.

### Analysis of the Primary Endpoint(s)

Primary Endpoint: TBI-CareQOL Caregiver Strain Short Form (SF). TBI-CareQOL Caregiver Strain assesses perceived feelings of feeling overwhelmed, stressed and “beat-down” related to the care partner role. Self-reported responses are on a Likert scale. This measure is scored on a *T* metric (M = 50; SD = 10). Higher scores indicate more strain.

Self-reported TBI-CareQOL Caregiver Strain (T score) will be used to test the effectiveness of the JITAI. Change from baseline to 6-months post-intervention will be compared between the two arms. Means and standard deviations of the within-person changes will be calculated. In addition, we will model the dependent variable (Caregiver Strain) using linear mixed effect model, where a random intercept will be included to account for repeated measurements from the same subject. We include in the model the following independent variables: the randomization arm, time and interaction between randomization arm and time, and baseline Caregiver Strain T scores. Key biologic variables such as age, sex, TBI severity, and co-morbidities will be among some of the factors explored as potential confounders. Differences between treatment arms can then be obtained by estimating and testing the corresponding parameters in the linear mixed effect model.

### Analysis of the Secondary Endpoint(s)

Secondary Endpoints:

- PROMIS Anxiety SF. PROMIS Anxiety assesses self-reported feelings of fear, anxiety and hyperarousal. Self-reported responses are on a Likert scale. This measure is scored on a *T* metric (M = 50; SD = 10). Higher scores indicate more anxiety. Administration time for this measure is ~1 minute.
- PROMIS Depression SF. PROMIS Depression assesses self-reported feelings of sadness and worthlessness. Self-reported responses are on a Likert scale. This measure is scored on a *T* metric (M = 50; SD = 10). Higher scores indicate more depression. Administration time for this measure is ~1 minute.

Self-reported PROMIS Anxiety (T score) or PROMIS Depression (T score) will be used to test the effectiveness of the JITAI. Change from baseline to 6-months post-intervention will be compared between the two arms. Means and standard deviations of the within-person changes will be calculated. In addition, we will model the dependent variable (Anxiety or Depression, respectively) using linear mixed effect model, where a random intercept will be included to account for repeated measurements from the same subject. We include in the model the following independent variables: the randomization arm, time and interaction between randomization arm and time, and baseline PROMIS Anxiety or PROMIS Depression T scores. Key biologic variables such as age, sex, TBI severity, and co-morbidities will be among some of the factors explored as potential confounders. Differences between treatment arms can then be obtained by estimating and testing the corresponding parameters in the linear mixed effect model.

### Baseline Descriptive Statistics

Care partners in each study group (JITAI and control) will be compared descriptively according to Consolidated Standards of Reporting Trials Guidelines.^35^

T tests/ANOVA will be used to examine group differences for continuous variables (e.g., age, HRQOL outcomes, time since injury, functional status of person with TBI). Chi-squared/Fisher exact tests will be used to examine group differences for categorical variables (e.g., sex, ethnicity, race, education, marital status, relationship to person with TBI, TBI severity).

# SUPPORTING DOCUMENTATION AND OPERATIONAL CONSIDERATIONS

## Regulatory, Ethical, and Study Oversight Considerations

### Informed Consent Process

#### Consent/assent and Other Informational Documents Provided to participants

The participant will be provided with a PDF of their certified consent form approved by the U-M IRBMED describing in detail the study intervention, study procedures, and risks. Informed consent will be obtained prior to the participant completing any study-related assessments. An example can be found in the U-M IRBMED application for this study.

#### Consent Procedures and Documentation

All consent procedures will be approved by the U-M IRBMED. Because participants do not need to come to a study site to participate in this study, informed consent may be conducted virtually (e.g., Zoom, Bluejeans, telephone, etc.) or in-person. Consent will be documented using the REDCap consent platform for all participants; participants will indicate their consent by entering their name and the date into the REDCap consent document. All individuals who are interested in participating in the study will be encouraged to ask questions about the study and their participation, and will be given as much time as needed to make a decision about participating. Participants will receive a pdf copy of their ‘signed’ consent.

A waiver of informed consent and HIPAA waiver is requested for eligibility screening for this study and for collection of medical record data for the individual with TBI that the participant (care partner) is caring for. The medical record data is needed to accurately capture injury severity, time since injury, etc.

### Confidentiality and Privacy

Participant confidentiality and privacy is strictly held in trust by the participating investigators, their staff, the independent safety monitor and funding agency. This confidentiality is extended to the data being collected as part of this study. Data that could be used to identify a specific study participant will be held in strict confidence within the research team.

All research activities will be conducted in as private a setting as possible. The study participant’s contact information will be securely stored at each site for internal use during the study. Each site will keep a master list of their site’s participant names and matching participant IDs. This master list will not be shared with the other site, and will be kept by site personnel in a password-protected file on a secure server or in a secure environment (e.g., locked cabinet, restricted access). No one other than the study team at the study site will have access to their site’s master list (except when needed for study monitoring). Informed consents will be stored in a study-specific database which contains only the informed consents and the informed consents for each site will only be available to their site’s study team members (except when needed for study monitoring). At the end of the study, all records will continue to be kept in a secure location for as long a period as dictated by the reviewing IRB, Institutional policies, or funding agency requirements.

No personal identifying information will appear on or with the participant data, where possible. Each participant will be assigned a participant ID by the study team to maintain confidentiality. The primary unique identifier is the study-assigned participant ID. The participant’s self-report data and CRF data will be identified with the participant ID. Additionally, an access code will be assigned to each participant which they will use to enroll in the CareQOL app. Participants will register their study Fitbit® on the Fitbit® app using a username and email address of their choice. The data collected on the Fitbit® will be linked to the CareQOL app by user authentication token, which is comprised of a random series of characters that is used to securely query for user activity data; no identifying information (including email address or Fitbit® user ID) is saved in the CareQOL app.

Study participant research data, which is for purposes of statistical analysis and scientific reporting, will be transmitted to and managed by the University of Michigan. Individual participants and their research data will be identified by a unique study identification number, where possible. The study data entry and study management systems (e.g., CareQOL app, REDCap, Qualtrics, M-Box, Fitbit®, etc.) used by clinical sites and by University of Michigan research staff are secured and password protected. At the end of the study, all study databases will be de-identified and archived at the University of Michigan.

Measures Taken to Ensure Confidentiality of Data Shared per the NIH Data Sharing Policies

It is NIH policy that the results and accomplishments of the activities that it funds should be made available to the public (see <https://grants.nih.gov/policy/sharing.htm>). The PI will ensure all mechanisms used to share data will include proper plans and safeguards for the protection of privacy, confidentiality, and security for data dissemination and reuse (e.g., all data will be thoroughly de-identified and will not be traceable to a specific study participant).

Certificate of Confidentiality

Participants in this study are covered under a NIH Certificate of Confidentiality (CoC). The CoC limits the disclosure of identifiable, sensitive information to a few instances: 1) if required by Federal, State, or local laws (e.g., as required by the Federal Food, Drug, and Cosmetic Act, or state laws requiring the reporting of communicable diseases to State and local health departments), excluding instances of disclosure in any Federal, State, or local civil, criminal, administrative, legislative, or other proceeding; 2) if necessary for the medical treatment of the individual to whom the information or document pertains and made with the consent of such individual; 3) if made with the consent of the individual to whom the information or document pertains; or 4) if made for the purposes of other scientific research that is in compliance with applicable Federal regulations governing the protection of human subjects in research. Participants will be notified in the informed consent that this study is covered by a CoC and will be informed of the protections and limitations to protections provided by the CoC.

### Future Use of Stored Specimens and Data

Data Retention

Research data and information will be retained for both study record-keeping purposes and for future research use. Any materials with identifiers (e.g., informed consents) will be stored in locked filing cabinet or on a secure server separate from coded study information.

Data from participants who withdraw from the study will be retained in the study database.

Study Record Keeping

During the study, we will maintain identifiable data to facilitate day-to-day study operations such as follow-up contact, distribution of study incentives, etc. These records may be destroyed after 7 years per U-M record keeping guidelines ([*https://az.research.umich.edu/medschool/guidance/record-keeping-guidelines*](https://az.research.umich.edu/medschool/guidance/record-keeping-guidelines)).

Data for Future Research Use

We will archive de-identified data for future research use. These data may be used for additional analyses related to the main study, new analyses, and in grant proposals for new research.

- The data will be stored on Michigan Medicine servers in a HIPAA-compliant electronic data capture system and/or statistical datasets that are stripped of identifiers.
- Access to the data will be at the discretion of the PI.
- Any data shared with collaborators and other researchers will be transferred using secure methods (e.g. MiShare, encryption, etc.)

### Key Roles and Study Governance

| **Principal Investigator** | **Independent Safety Monitor** |
| --- | --- |
| Noelle Carlozzi, PhD, Associate Professor, Dept. of Physical Medicine & Rehabilitation, Medical School | Mary Janevic, MPH, PHD, Associate Research Scientist, Health Behavior/Health Education, School of Public Health |
| University of Michigan | University of Michigan |
| 2800 Plymouth Rd., NCRC Building 14, Ann Arbor, MI 48109-2800 | 2817 SPH 1  Ann Arbor, MI 48109-2029 |
| 734-763-8917 | 734-647-3194 |
| Carlozzi@med.umich.edu | mjanevic@umich.edu |

### Safety Oversight

Mary Janevic, MPH, PhD, will serve as the Independent Safety Monitor (ISM) for this study. Dr. Janevic is an Associate Research Scientist in Health Behavior/Health Education at the U-M School of Public Health and is a faculty member of the University of Michigan’s Center for Managing Chronic Disease. She is not a member of the study team and is available to review and recommend appropriate action over the duration of this five-year study. Her ISM responsibilities include:

- Reviewing the research protocol, informed consent documents and plans for data safety and monitoring.
- Recommending subject recruitment be initiated after the receipt of a satisfactory protocol.
- Evaluating the progress of the trial, including annual assessments of data quality and timeliness, recruitment, accrual and retention, participant risk versus benefit, overall performance of the trial and other factors that can affect outcomes.
- Protecting the safety of study participants.
- Considering factors external to the study when relevant information becomes available, such as scientific or therapeutic developments that may have an impact on the safety of the participants or the ethics of the trial.
- Reviewing study performance, making recommendations and assisting in the resolution of problems reported by the PI.
- Ensuring the confidentiality of study data.
- Reporting on the safety and progress of the trial annually.
- Making recommendations to the PI and IRB about the continuation, termination or modifications of the trial based on the observed beneficial or adverse effects of the treatment.

The PI will meet with the ISM prior to the start of participant recruitment and annually throughout the duration of the study, coinciding with the IRB scheduled continuing review. Additionally, the ISM will be available and consulted if greater than expected safety concerns or other issues arise. The ISM will prepare an annual report for the PI. The PI will be responsible for submitting this annual report to the U-M IRBMED and NINR.

### Quality Assurance and Quality Control

QA and QC practices will be the responsibility of all team members. Training and regular meetings (held remotely or in-person) will be conducted to monitor and facilitate quality data collection.

**Training:**

All site investigators and study staff will be trained on the project protocol, project data collection systems and other study-related topics by the principal investigator and the project manager prior to beginning data collection. The standardized training will be conducted at all sites prior to them enrolling participants in the study. Additionally, the project manager, in cooperation with the PI and the data manager, will develop a detailed manual of procedures, case report forms and other related study materials. The manual of procedures will provide step-by-step instructions on the conduct of the trial, including how to use and troubleshoot all of the data collection platforms. The manual of procedures and other study documents will be reviewed in detail with the data collectors at each site as part of the training, and all materials will be available on the study-specific shared MBox site for access at any time. Study staff will be encouraged to use the step-by-step instructions when conducting participant visits to ensure that they are adhering to the study protocol. Ongoing training will occur via regular conference calls and during site visits. Additionally, any staff who join the team after the project starts will be required to complete the standardized training prior to enrolling participants. Training will be documented in the electronic regulatory binder.

**Meetings:**

- **Full Team (U-M and BCM):** Full team meetings will be held prior to enrolling participants (study launch) and yearly thereafter to focus/re-focus the project goals, summarize challenges and successes, and participate in applicable trainings
- **Investigator Meetings** – Meetings (remote or in-person) with the study investigators will be held monthly to discuss study activities, progress and troubleshoot any issues. Other project staff will attend as needed.
- **Project Staff Meetings** – The Project Manager will hold monthly meetings after data collection begins with the project staff. These meetings will be focused on tracking recruitment efforts, data collection (including data management and clearing of queries), and discussion of protocol deviations or adverse events. Investigators may attend as needed.
- **Data Management** **Team** – The PI, study statistician, and Data & Project Managers will meet regularly throughout the project; these meetings will occur more often as the study launches, and then less frequently thereafter to develop and uphold the data management plan (from collection through analysis). Other attendees (e.g. investigators, U-M/BCM staff, etc.) may be included as needed
- **Site Visits:** Annual visits (virtual or in-person) by the PI and the Project Manager will be conducted at both data collection sites to review study regulatory materials, informed consents, data, procedures and compliance to the study protocol.

### Data Handling and Record Keeping

#### Data Collection and Management Responsibilities

This project uses multiple electronic data capture and management platforms (e.g., REDCap, CareQOL, Qualtrics, Fitbit®, U-M server, Google Cloud, AWS Cloud). All platforms are designed for human subjects research and comply with federal and local data and information security practices. Each site will use the same platforms.

Data collection is the responsibility of the site study staff under the direction of the Project Manager and site PI. All data are entered or captured on the platforms by study staff or directly by participants (e.g. sleep/activity data, daily EMAs etc.). Any study documents completed on paper will be completed legibly and in ink.

The Project and Data Manager and the PI will directly oversee the QA/QC activities using standard operating procedures, checklists, and built-in and study-specific data validation rules to uphold data integrity. Routine data backups will be part of this process.

Annual site visits will be made by the U-M PI and project manager will be conducted at U-M and BCM and are described below:

- Auditing Selected Cases for Compliance with IRB Requirements. These annual audits will include a random review of 20% of the cases that have been collected since the previous review.
- Conformance with Informed Consent Requirements. As part of these annual assessments, the project manager and PI will also review all consent forms for participants that have been seen since the previous review.
- Verification of Source Documents. These annual audits will also include a verification of all source documents at both sites.
- Investigator Compliance. We will review the site regulatory binder to ensure that all study investigators have updated CVs and documentation of human subjects’ training.

To document adherence to this plan we will maintain a monitoring log and create accompanying monitoring reports for each review. The monitoring log will be kept in the electronic regulatory binder.

The data manager will be responsible for setting up, monitoring and maintaining the data collection systems for both sites, and collaborating with the Project Manager on the initial implementation checks of data quality. Initially, data from the mobile app will be reviewed following the run-in period from the first 5 participants to ensure that daily data is being appropriately collected. We will continue to monitor the same 5 participants throughout the completion of the study (including the end of month surveys during the home monitoring period and the post-home monitoring surveys). We will evaluate procedures to ensure that data transfers at all points are accurate. Review of the data quality will be conducted by the data manager and reviewed by the study statistician and PI. Irregularities or problems detected will be discussed with the study team and addressed. Following any needed adjustments or corrections to study procedures, subsequent data quality as described above will be monitored for the next 5 participants and then by random inspection of the data independently by the data manager, statistician and PI. Quarterly audits will confirm proper data transfers and downloads, and include checks for missing and/or out-of-range data, logic errors, etc. Quality control and reliability of the data will be discussed at regular team meetings.

#### Study Records Retention

Study records will be retained according to University of Michigan guidance for research records retention involving health-related data ([*https://az.research.umich.edu/medschool/guidance/record-keeping-guidelines*](https://az.research.umich.edu/medschool/guidance/record-keeping-guidelines)).

### OTHER REPORTABLE INCIDENTS & OCCURRENCES (ORIOS)

Both sites will adhere to IRBMED’s Multi-Site Research Reporting Plan (see Appendix 1) for ORIOs, including UaPs and Protocol Deviations. Briefly, this plan requires reporting of events to IRBMED within 7 days of the study team’s awareness of said event. Refer to Appendix 1 for list of reportable events.

**Unanticipated Problems:**

An Unanticipated Problem Involving Risks to Subjects or Others (UaP) is an actual incident, experience or outcome that warrants consideration of substantive changes in the research protocol or informed consent process/document or other corrective actions in order to protect the safety, welfare or rights of subjects or others. The following criteria must be met:

- Unexpected (in terms of nature, severity, or frequency) given (a) the research procedures that are described in the protocol-related documents, such as the Institutional Review Board (IRB)-approved research protocol and informed consent document; and (b) the characteristics of the participant population being studied;
- Related or possibly related to participation in the research (possibly related means there is a reasonable possibility that the incident, experience, or outcome may have been caused by the procedures involved in the research); and
- Suggests that the research places subject(s) or other at a greater risk of harm (including physical, psychological, economic, or social harm) than was previously known or recognized.

Unanticipated problems will be reported by the principal investigator to the U-M IRBMED per their standard reporting timeline. See: <https://az.research.umich.edu/medschool/guidance/unanticipated-problems-involving-risks-subjects-or-others>

Sites will report study-related unanticipated problems using the unanticipated problem form in the REDCap study database. Unanticipated problems will be discussed at investigator and project staff meetings.

The study ISM will review all UaPs at least annually throughout the course of the study. They will be summarized in the ISM report that is submitted to the U-M IRBMED and NINR at annual review and in other ISM reports (if applicable).

**Protocol Deviations:**

This protocol uses the University of Michigan definition of a protocol deviation (<https://az.research.umich.edu/medschool/glossary/deviation>) which defines a protocol deviation as “an incident involving non-compliance with the protocol, but one that does not have a significant effect on the subject’s rights, safety or welfare, and/or on the integrity of the data. Deviations may result from the action of the participant, researcher or staff.”

We will follow the U-M IRBMED reporting guidance for protocol deviations found here <https://az.research.umich.edu/medschool/guidance/other-reportable-information-or-occurrence-orio>.

Reportable protocol deviations will be reported and tracked in the REDCap database and will be discussed at investigator and project staff meetings.

The following events will not be reported as protocol deviations:

- A protocol deviation will not be reported for participants who skip/do not complete study survey questions or entire surveys (like end of month assessments or post-intervention surveys). Participants can decline to answer any survey question for any reason. We expect in an intensive, long duration protocol like this that participants may miss some assessments.
- A protocol deviation will not be reported for out-of-window assessments. Use of this data will be assessed by the study investigators for each study analysis; some analyses may need tighter compliance to the assessment window while others will not.
- A protocol deviation will not be reported for participants who miss/do not complete the EMA assessments or do not wear/miss uploading Fitbit® data during the home-monitoring period. For example, we expect that there may be instances where participants are unable to complete the EMA assessments (for example, they are somewhere where it is inconvenient for them to answer) or forget to put on or sync the Fitbit®.
- A protocol deviation will not be reported for participants who decline the participant payment. It has been our experience that some research participants do not wish to receive payment for their participation in research studies.

Reportable protocol deviations will be reviewed at the project staff and investigator meetings, and summarized in the ISM report that it submitted to the U-M IRBMED and NINR at annual review (coinciding with the scheduled continuing review) and in other ISM reports (if applicable).

### Publication and Data Sharing Policy

The University of Michigan (U-M) is committed to the Data Sharing Policies specified by the study funder. The Principal Investigator and her collaborators will make unique research resources readily available for research purposes to individuals within the scientific community after publication. U-M has previously used a variety of means as appropriate and expeditious to share data resulting from sponsored projects with research colleagues, such as depositing data into secure web-accessible data warehouses or arranging distribution of data, and protocols to other researchers using established mechanisms and repositories.

U-M will assure the timely release and sharing of data no later than the acceptance for publication of the main findings from the final dataset and will protect the rights and privacy of human subjects who participate in research by redacting all identifiers, and adopting other strategies to minimize risks of unauthorized disclosure of personal identifiers in accordance with authorization and consent documents. U-M agrees that data sharing is essential for expedited translation of research results into knowledge, products, and procedures to improve human health. To enable efficient data sharing, the U-M Project Manager will coordinate requests for data and maintain documentation for requests and distributions. The University has an established Institutional Data Use Agreement that can easily be adapted and deployed.

This study will comply with the National Institutes of Health (NIH) Public Access Policy, which ensures that the public has access to the published results of NIH funded research. It requires scientists to submit final peer-reviewed journal manuscripts that arise from NIH funds to the digital archive PubMed Central upon acceptance for publication.

This study will also comply with the NIH Data Sharing Policy and Policy on the Dissemination of NIH-Funded Clinical Trial Information and the Clinical Trials Registration and Results Information Submission rule. As such, this trial will be registered at ClinicalTrials.gov, and results information from this trial will be submitted to ClinicalTrials.gov.

# REFERENCES

1. Rothing M, Malterud K, Frich JC. Caregiver roles in families affected by Huntington's disease: a qualitative interview study. *Scand J Caring Sci.* 2013.

2. Verhaeghe S, Defloor T, Grypdonck M. Stress and coping among families of patients with traumatic brain injury: a review of the literature. *J Clin Nurs.* 2005;14(8):1004-1012.

3. Carlozzi NE, Brickell TA, French LM, et al. Caring for our wounded warriors: A qualitative examination of health-related quality of life in caregivers of individuals with military-related traumatic brain injury. *Journal of Rehabilitation Research & Development.* 2016;53(6):669-680.

4. Carlozzi NE, Hanks R, Lange RT, et al. Understanding Health-related Quality of Life in Caregivers of Civilians and Service Members/Veterans With Traumatic Brain Injury: Establishing the Reliability and Validity of PROMIS Mental Health Measures. *Arch Phys Med Rehabil.* 2018.

5. Carlozzi NE, Ianni PA, Lange RT, et al. Understanding health-related quality of life of caregivers of civilians and service members/veterans with Traumatic Brain Injury: Establishing the reliability and validity of PROMIS social health measures. *Arch Phys Med Rehabil.* 2018.

6. Carlozzi NE, Ianni PA, Tulsky DS, et al. Understanding Health-Related Quality of Life in Caregivers of Civilians and Service Members/Veterans With Traumatic Brain Injury: Establishing the Reliability and Validity of PROMIS Fatigue and Sleep Disturbance Item Banks. *Arch Phys Med Rehabil.* 2018.

7. Carlozzi NE, Kallen MA, Hanks R, et al. The TBI-CareQOL Measurement System: Development and Preliminary Validation of Health-Related Quality of Life Measures for Caregivers of Civilians and Service Members/Veterans With Traumatic Brain Injury. *Arch Phys Med Rehabil.* 2018.

8. Carlozzi NE, Kallen MA, Hanks R, et al. The development of a new computer adaptive test to evaluate feelings of being trapped in caregivers of individuals with traumatic brain injury: TBI-CareQOL Feeling Trapped Item Bank. *Arch Phys Med Rehab.* 2018.

9. Carlozzi NE, Kallen MA, Ianni PA, et al. The Development of a New Computer-Adaptive Test to Evaluate Strain in Caregivers of Individuals With TBI: TBI-CareQOL Caregiver Strain. *Arch Phys Med Rehabil.* 2018.

10. Carlozzi NE, Kallen MA, Ianni PA, et al. The development of a two new computer adaptive tests to evaluate feelings of loss in caregivers of individuals with traumatic brain injury: TBI-CareQOL Feelings of Loss-Self and Feelings of Loss-Person with Traumatic Brain Injury. *Arch Phys Med Rehabil.* 2018.

11. Carlozzi NE, Kallen MA, Sander AM, et al. The development of a new computer adaptive test to evaluate anxiety in caregivers of individuals with traumatic brain injury: TBI-CareQOL Caregiver-Specific Anxiety. *Arch Phys Med Rehabil.* 2018.

12. Carlozzi NE, Kratz AL, Sander AM, et al. Health-related quality of life in caregivers of individuals with traumatic brain injury: development of a conceptual model. *Archives of Physical Medicine & Rehabilitation.* 2015;96(1):105-113.

13. Carlozzi NE, Lange RT, French LM, Sander AM, Freedman J, Brickell TA. A Latent Content Analysis of Barriers and Supports to Healthcare: Perspectives From Caregivers of Service Members and Veterans With Military-Related Traumatic Brain Injury. *J Head Trauma Rehabil.* 2018.

14. Carlozzi NE, Lange RT, French LM, et al. Understanding Health-Related Quality of Life in Caregivers of Civilians and Service Members/Veterans With TBI: Reliability and Validity Data for the TBI-CareQOL Measurement System. *Arch Phys Med Rehabil.* 2018.

15. Song Y, Keatley LR, Middleton PG. Methicillin-resistant Staphylococcus aureus in health-care workers with cystic fibrosis in Sydney. *Respirol Case Rep.* 2018;6(9):e00378.

16. Kratz AL, Sander AM, Brickell TA, Lange RT, Carlozzi NE. Traumatic brain injury caregivers: A qualitative analysis of spouse and parent perspectives on quality of life. *Neuropsychol Rehabil.* 2017;27(1):16-37.

17. Sander AM, Hanks RA, Ianni PA, et al. Sociocultural factors influencing caregiver appraisals following traumatic brain injury. *Arch Phys Med Rehab.* 2018.

18. Carlozzi NE, Kallen MA, Brickell TA, et al. Measuring emotional suppression in caregivers of adults with traumatic brain injury. *Rehabil Psychol.* 2019.

19. Sander AM, Boileau NR, Hanks RA, Tulsky DS, Carlozzi NE. Emotional Suppression and Hypervigilance in Military Caregivers: Relationship to Negative and Positive Affect. *J Head Trauma Rehabil.* 2020;35(1):E10-E20.

20. Carlozzi NE, Lange RT, Kallen MA, et al. Assessing vigilance in caregivers after traumatic brain injury: TBI-CareQOL Caregiver Vigilance. *Rehabil Psychol.* 2020.

21. Carlozzi NE, Lange RT, French LM, et al. TBI-CareQOL military health care frustration in caregivers of service members/veterans with traumatic brain injury. *Rehabil Psychol.* 2020.

22. Carlozzi NE, Lange RT, Boileau NR, et al. TBI-CareQOL family disruption: Family disruption in caregivers of persons with TBI. *Rehabil Psychol.* 2019.

23. Carlozzi NE, Boileau NR, Kallen MA, et al. Reliability and validity data to support the clinical utility of the Traumatic Brain Injury Caregiver Quality of Life (TBI-CareQOL). *Rehabil Psychol.* 2019.

24. Hanks RA, Boileau NR, Norman AL, Nakase-Richardson R, Mariouw KH, Carlozzi NE. Spirituality and outcomes in caregivers of persons with traumatic brain injury (TBI). *Rehabil Psychol.* 2020.

25. Brickell TA, Cotner BA, French LM, et al. Severity of military traumatic brain injury influences caregiver health-related quality of life. *Rehabil Psychol.* 2020.

26. Kratz AL, Boileau NR, Sander AM, et al. Do emotional distress and functional problems in persons with traumatic brain injury contribute to perceived sleep-related impairment in caregivers? *Rehabil Psychol.* 2020.

27. Raad JH, Tulsky D, Lange RT, et al. Establishing the Factor Structure of a Health-Related Quality of Life Measurement System for Caregivers of Persons Living with Traumatic Brain Injury. *Arch Phys Med Rehabil.* 2020.

28. Carlozzi NE, Boileau NR, Hanks RA, Sander AM, Nakase-Richardson R, Massengale JP. Sleep impairment is related to health-related quality of life among caregivers of lower-functioning traumatic brain injury survivors. *Rehabil Psychol.* In Press.

29. Kalmbach DA, Fang Y, Arbedt JT, et al. Effects of Sleep, Physical Activity, and Shift Work on Daily Mood: a Prospective Mobile Monitoring Study of Medical Interns. *J Gen Intern Med.* 2018;33(6):914-920.

30. Nahum-Shani I, Smith SN, Spring BJ, et al. Just-in-Time Adaptive Interventions (JITAIs) in Mobile Health: Key Components and Design Principles for Ongoing Health Behavior Support. *Ann Behav Med.* 2018;52(6):446-462.

31. Fitzmaurice GM, Laird NM, Ware JH. *Applied longitudinal analysis.* Vol 998. Boston, MA: John Wiley & Sons; 2012.

32. Powell JM, Fraser R, Brockway JA, Temkin N, Bell KR. A Telehealth Approach to Caregiver Self-Management Following Traumatic Brain Injury: A Randomized Controlled Trial. *J Head Trauma Rehabil.* 2016;31(3):180-190.

33. Holmen H, Torbjornsen A, Wahl AK, et al. A Mobile Health Intervention for Self-Management and Lifestyle Change for Persons With Type 2 Diabetes, Part 2: One-Year Results From the Norwegian Randomized Controlled Trial RENEWING HEALTH. *JMIR Mhealth Uhealth.* 2014;2(4):e57.

34. Whitehead L, Seaton P. The Effectiveness of Self-Management Mobile Phone and Tablet Apps in Long-term Condition Management: A Systematic Review. *Journal of medical Internet research.* 2016;18(5):e97.

35. Schulz KF, Altman DG, Moher D. CONSORT 2010 statement: Updated guidelines for reporting parallel group randomised trials. *J Pharmacol Pharmacother.* 2010;1(2):100-107.

# APPENDIX 1: MULTI-SITE RESEARCH REPORTING PLAN

**REPORTABLE WITHIN 7 DAYS**

The following types of events must be reported to the lead site (University of Michigan) study team within 7 calendar days of *becoming aware of the event*.

**Unanticipated Problems Involving Risks to Human Subjects or Others:** an actual incident, experience, or outcome that warrant consideration of substantive changes in the research protocol or informed consent process/document or other corrective actions in order to protect the safety, welfare, or rights of subjects or others. The following criteria must be met:

1. Unexpected (in terms of nature, severity, or frequency) given (a) the research procedures that are described in the protocol-related documents, such as the IRB-approved research protocol and informed consent document; and (b) the characteristics of the subject population being studied

2. Related or possibly related to participation in the research (possibly related means there is a reasonable possibility that the incident, experience, or outcome may have been caused by the procedures involved in the research);

3. Suggests that the research places subject(s) or others at a greater risk of harm (including physical, psychological, economic, or social harm) than was previously known or recognized.

**Non-compliance:** The failure of a person or organization to act in accordance with the requirements of a law, regulation, policy, or the requirements and/or determination of an IRB. Major protocol deviations that may adversely impact safety of participants, or impact integrity/validity of the data are considered non-compliance (such as dosage errors/intervention errors, consent process deviations, deliberate procedural deviations, and accidental procedural deviations)

**Continuing non-compliance:** Noncompliance that recurs after an investigator has been notified of a similar or related noncompliance concern pertaining to one or more protocols.

**Serious non-compliance:** Non-compliance that, in the judgment of the IRB, materially increases the risks or causes substantive harm to research participants or materially compromises the rights or welfare of participants including consideration of the following:

1. Harm to participants;

2. Exposure of participants to a significant risk of substantive harm;

3. Compromised privacy and confidentiality of participants;

4. Willful or knowing research misconduct on the part of the investigator;

5. A violation of ethical principles for human research; or

6. Damage caused to the scientific integrity of the data collected.

**Complaints:** Complaints from any individual related to participant safety, study conduct, or study related materials.

**Accident/Incident:** Accidents/Incidents involving participants, their data, biospecimens or facilities associated with the research (e.g., breach of confidentiality, loss of research data or biospecimens).

**Subject Incarceration:** Incarceration of a participant when the research was not previously approved for the enrollment of prisoners under Subpart C and the investigator believes it is in the best interest of the subject to remain on the study.

**Oversight Reports:** Reports of internal or external audits; study holds or suspensions that are not built into the study design and affect the local site only. Reports of monitoring (such as Data Safety Monitoring) outcomes that have concerns of subject safety or suggested revision of study materials.

**Subject Withdrawal:** Withdrawal due to safety reasons.

**Pertinent publication/public announcement:** Information affecting the risk/benefit ration of the study or information affecting subjects’ willingness to participate in the research.

**IRB Approval Lapse:** Report of any study activity during the lapse in approval (this can happen if a site does not get information to lead site in time for the submission of the continuing review).

**REPORTABLE AT CONTINUING REVIEW**

The following types of events must be reported to the lead site (University of Michigan) study team at the next scheduled continuing review.

**Site Status Reports:** Site enrollment closed and/or completed interaction/intervention notifications without safety or regulatory concerns.

**Subject Withdrawal:** Withdrawal of a subject due to PI discretion, subject discretion/request or other reasons, such as meeting protocol stopping rules.
